# Modulation of TvRAD51 Recombinase in *Trichomonas vaginalis* by Zinc and Cadmium as a Potential Mechanism for Genotoxic Stress Response

**DOI:** 10.3390/pathogens14060565

**Published:** 2025-06-05

**Authors:** Jonathan Puente-Rivera, José Jesús Flores-Vega, Marcos Morales-Reyna, Elisa Elvira Figueroa-Angulo, Yussel Pérez-Navarro, Alfonso Salgado-Aguayo, Ángeles Carlos-Reyes, Maria Elizbeth Alvarez-Sánchez

**Affiliations:** 1 Laboratorio de Patogénesis Celular y Molecular Humana y Veterinaria, Posgrado en Ciencias Genómicas, Universidad Autónoma de la Ciudad de México (UACM), San Lorenzo 290, Col. Del Valle, Ciudad de México 03100, Mexico; jo_puenter@hotmail.com (J.P.-R.);; 2 División de Investigación, Hospital Juárez de México, Instituto Politécnico Nacional 5160, Col. Magdalena de las Salinas, Ciudad de México 07760, Mexico; 3 Laboratorio de Investigación en Enfermedades Reumáticas, Instituto Nacional de Enfermedades Respiratorias, Calzada de Tlalpan 4502, Belisario Domínguez Secc 16, Tlalpan, Ciudad de México 14080, Mexico; 4 Laboratorio de Onco-Inmunobiología, Instituto Nacional de Enfermedades Respiratorias, Calzada De Tlalpan 4502, Belisario Domínguez Secc 16, Tlalpan, Ciudad de México 14080, Mexico

**Keywords:** *Trichomonas vaginalis*, DNA damage, TvRAD51, zinc, cadmium

## Abstract

*Trichomonas vaginalis*, the protozoan responsible for trichomoniasis, encounters fluctuating levels of metal cations in the male urogenital tract, notably zinc (Zn^2+^) and cadmium (Cd^2+^), which may induce genotoxic stress. While zinc is a key physiological component of the male reproductive tract, both Zn^2+^ and Cd^2+^ can become genotoxic at elevated concentrations. However, their effect on DNA repair mechanisms in *T. vaginalis* remains poorly understood. This study characterizes, for the first time, the expression and modulation of the recombinase TvRAD51, a homologous recombination (HR) key enzyme, in response to UV irradiation and sublethal concentrations of Zn^2+^ (1.6 mM) and Cd^2+^ (0.1 mM). In silico analyses confirmed the presence and conserved structure of the tvrad51 gene and its interaction with HR-related proteins, such as TvBLM and TvBRCA2. Quantitative RT-PCR, Western blot, and immunofluorescence assays revealed that TvRAD51 is upregulated at both transcript and protein levels following UV- and cation-induced DNA damage, with distinct temporal expression patterns for Zn^2+^ and Cd^2+^ exposure. Notably, TvRAD51 showed nuclear localization at early time points post-exposure, suggesting active participation in DNA repair processes. These findings demonstrate that TvRAD51 is a central component of the genotoxic stress response in *T. vaginalis*, potentially contributing to parasite survival and adaptation in hostile environments through homologous recombination repair pathways.

## 1. Introduction

*Trichomonas vaginalis* is the causative agent of trichomoniasis, the most prevalent non-viral sexually transmitted infection (ITS) worldwide. This cosmopolitan parasite significantly impacts the sexual health of both men and women [1]. Trichomoniasis affected 156 million people in 2023, according to official data from the World Health Organization [2]. This parasite can adapt to and colonize prostatic tissue even under the adverse microenvironmental conditions presented by prostatic fluid, particularly with Zn^2+^ concentrations ranging from 4 to 7 mM. Despite this, male trichomoniasis has been relatively understudied, and the pathogenic mechanisms of *T. vaginalis* and the factors leading to disease remain largely unknown [3]. The zinc cation (Zn^2+^) plays a fundamental role in the male reproductive tract, functioning as a natural antimicrobial agent against bacteria, viruses, and certain fungi, and it is particularly critical in the prostate, where it helps prevent pathogen invasion and supports male fertility [4].

It has been reported that Zn^2+^ concentrations of 1.6 mM are trichomonicidal, and a zinc-related metalloproteinase, TvMP50, has been identified in *T. vaginalis*, found exclusively in male samples [5]. Recent studies have suggested that Zn^2+^, an essential cofactor in many enzymes and proteins, plays a role in regulating virulence and oxidative stress responses in *T. vaginalis* [6]. However, the impact of cadmium, a heavy metal with well-known genotoxic properties [7] on the expression of DNA repair proteins such as RAD51, has not been thoroughly investigated in this organism.

The recombinase RAD51 is a key enzyme in DNA repair through homologous recombination (HR), a process crucial for maintaining genome integrity in response to double-strand breaks [8]. The expression and activity of RAD51 have been well-documented in various organisms under genotoxic stress, including exposure to heavy metals such as cadmium (Cd^2+^) [9] or treatment in diseases like cancer [10]. These metals, while essential in small amounts like Zn^2+^, could be toxic and mutagenic at sublethal concentrations, highlighting the importance of DNA repair mechanisms in cellular survival under these conditions. Recombination events and the expression and involvement of the RAD51 protein have been observed in different parasites, particularly protozoans. The first study on HR in *Entamoeba histolytica* examined this organism’s response to UV-C radiation, a known DNA damage inducer, and observed central HR protein signature.

Although cellular survival was unaffected, there was a differential expression response to irradiation, with EhRAD51 expression (both mRNA and protein) peaking rapidly and forming nuclear foci in response to DNA damage [11]. In *Leishmania infantum*, the protein LiRAD51 was found to be highly expressed following exposure to bleomycin. Both LiRAD51 and LiBRCA2 localize to nuclear foci; however, LiRAD51 fails to localize to the nucleus in cells deficient in LiBRCA2 [12]. Regarding *Plasmodium falciparum*, PfRad51 has been shown to hydrolyze ATP and efficiently facilitate DNA strand exchange; although PfRAD51 requires ATP binding, it does not require hydrolysis to catalyze DNA strand exchange in vitro [13], similar to the human RAD51 protein, and the mutational analysis of the ATP-binding motif in PfRad51 (PfRAD51K143R) demonstrated a significant impact on the parasite and consequently the reduction in infection in mice [14].

Recent in silico evidence suggests the potential expression of proteins involved in the functional machinery of HR in *T. vaginalis*, including the key recombinase RAD51, with biological functions in this and other parasitic protozoa beyond DNA damage repair [15]. In this study, to our knowledge, the transcriptional expression and protein-level abundance of RAD51 in *T. vaginalis* exposed to UV-treatment and sublethal concentrations of Zn^2+^ (1.6 mM) and Cd^2+^ (0.1 mM) were evaluated for first time. In this context, understanding how *T. vaginalis* responds to metal-induced genotoxic stress is crucial, particularly given the high concentrations of Zn^2+^ and the presence of Cd^2+^ in the male urogenital tract. Despite the known roles of RAD51 in homologous recombination and DNA repair in other protozoan parasites, its regulation and function in *T. vaginalis* remain unexplored. This study provides the first experimental evidence of the expression, regulation, and subcellular localization of TvRAD51 in response to UV-induced DNA damage and exposure to sublethal concentrations of Zn^2+^ and Cd^2+^. Using a combination of in silico analysis, quantitative RT-PCR, Western blotting, and immunofluorescence, we aim to (1) characterize the *tvrad51* gene and its predicted interactions with other DNA repair proteins, (2) evaluate the gene and protein expression profiles of TvRAD51 under genotoxic conditions, and (3) determine its spatial localization within the parasite. These findings offer new insights into the DNA damage response of *T. vaginalis*, suggesting that TvRAD51 may be a key player in genome stability and parasite survival under metal-induced stress—an area previously uncharacterized in this organism.

## 2. Materials and Methods

### 2.1. In Silico TvRAD51 Analysis

The analysis of the *tvrad51* gene was performed using sequences obtained from the *T. vaginalis* G3 database TrichDB v.68 (http://trichdb.org/trichdb/ (accessed on 28 January 2025)) and reported ESTs from genes that codified for homologous proteins of HR machinery in the parasite as accession numbers TvRAD51 (TVAG_204070), TvXRCC3 (TVAG_144570), TvBRCA2 (TVAG_473090), TvRAD54 (TVAG_441050), TvRAD50 (TVAG_332600), and TvMRE11 (TVAG_098295).

### 2.2. Sequence, Phylogenetic, and Tertiary Structure Prediction of TvRAD51

The predicted amino acid sequence corresponding to RAD51 recombinase, dubbed TvRAD51, was compared with homologous proteins from other organisms by BLASTp and aligned with by ClustalW (http://workbench.sdsc.edu/ (accessed on 2 February 2025)). A phylogenetic tree was constructed using the neighbor-joining method with Bioedit software v7.2 6.1 (http://www.mbio.ncsu.edu/bioedit/page2.html/ (accessed on 5 February 2025 )). Bootstrap proportions were used to assess the robustness of the tree with 100 bootstrap replications. The two and three-dimensional homology modeling of TvRAD51 was performed using SWISS model (https://swissmodel.expasy.org (accessed on 3 March 2025)) and was based on the proposed model using the crystal structure of RAD51 homolog from *Homo sapiens* (pdb: 5JZC) as a template obtained from the Protein Data Bank (http://www.rscb.org/ (accessed on 3 March 2025)) and was also predicted using AlphaFold (https://alphafoldserver.com/ (accessed on 3 March 2025)), a state-of-the-art deep learning algorithm for protein structure prediction. The TVAG_2040270 (A2FXT7) sequence was input into the AlphaFold pipeline, which generated multiple predicted models. The highest-confidence model was selected based on predicted Local Distance Difference Test (pLDDT) scores. The visualization and structural analysis of the predicted model were performed using the molecular graphics software of same the platform. The predicted structure was further validated through a comparison with known homologous structures and the assessment of the expected position error to ensure accuracy of key functional domains. The subsequent analysis, preparation, and visualization of the model were performed using SWISS model Expasy.

### 2.3. Prediction of Interaction Analysis

Functional protein association network analysis was performed using the Search Tool for the Retrieval of Interacting Genes/Proteins STRING database v12.0 https://string-db.org/ (accessed on 18 March 2025) [16] with TVAG_204070 as query. The model includes the proteins codified by genes, the expression of which was corroborated by EST analysis, as described in the in silico analysis.

### 2.4. Parasites Culture, UV Irradiation, and Cations Treatments

For the parasite culture, the *T. vaginalis* CNCD147 isolate was cultivated to the mid-logarithmic phase in Diamond’s trypticase-yeast extract-maltose (TYM) medium (pH 6.2), supplemented with 10% (*v*/*v*) heat-inactivated horse serum [17]. In all assays, the final concentration of Zn^2+^ (Sigma-Aldrich, Co., St. Louis, MO, USA) was 1.6 mM [18] and the concentration of Cd^2+^ was 0.1 mM [19]. Parasite viability for each condition was confirmed using the trypan blue exclusion test, with 99 to 100% viability and no *Mycoplasma* contamination detected. For UV-C light irradiation, 10 × 10^6^ parasites from the same isolate were grown into plate dishes and incubated at 37 °C for 30 min. Medium and floating cells were discarded, and adhered trophozoites were irradiated with 254 nm UV-C light at 150 J/m^2^ in a UV Stratalinker 1800 device (Stratagene, La Jolla, CA, USA) [20]. After genotoxic insult, parasites were incubated in fresh TYM medium for 10, 60, and 120 min to be used in the experiments. Non-irradiated cells (-UV) or treated without cations (-Cd^2+^-Zn^2+^) were used as a control (CTRL) in all experiments. Cell viability was monitored by microscopy using a trypan blue dye exclusion test. Each biological assay was performed twice by technical triplicate.

### 2.5. RNA Isolation and End-Point PCR

The total RNA was extracted from 1 × 10^7^ parasites grown with or without Zn^2+^, Cd^2+^, and UV treatment using TRIzol (Invitrogen, Life Technologies, Carlsbad, CA, USA) according to the manufacturer’s instructions. For qRT-PCR, total RNA (1 μg) was reverse transcribed using a Superscript II reverse transcriptase kit (Invitrogen, Thermo Scientifc, Waltham, MA, USA) and oligo (dT20) primer (Invitrogen). Reactions contained 1 μg of cDNA from each condition as a template, 10 pmol of each primer specific to the *tvrad51* gene forward primer (5′-CGACTGGGACTGGGCTTTGAAACTCG-3′), and reverse primer (5′-TCAAAGTACTCTTGAAGC-3′). As a control, a 112 bp intern region of the β-tubulin fragment gene was amplified using the BTUB9 forward primer (5′-CATTGATAACGAAGCTCCTTTACGAT-3′) and BTUB2 reverse primer (5′-GCATGTTGTGCCGGACATAACCAT-3′) [21]. The cycling conditions were 10 min of polymerase activation at 95 °C followed by 35 cycles at 95 °C for 30 s, 48 °C for 15 s, and 72 °C for 30 s in a GeneAmp© PCR system 9700 (AppliedBiosystems Waltham, MA, USA). β-tubulin mRNA levels were used for normalization. Three independent biological assays were performed (Supplementary Figure S1).

### 2.6. Western Blot Assays

For Western blot (WB) assays, we employed a method previously described by [22]. Proteins of each condition (CTRL, +UV, +Zn^2+^ and +Cd^2+^ treatments) were quantified by the Bradford assay (Bio-Rad, Hercules, CA, USA) and 200 µg of total protein was separated by SDS-PAGE and transferred onto nitrocellulose (NC) membranes (Bio-Rad) and subsequently blocked with PBS1x non-fat dry milk (2.5%). The membranes were then incubated with mouse anti-human RAD51 (1:1000 dilution) (Santa Cruz Biotechnology, Santa Cruz, CA, USA) and mouse anti human α-tubulin (1: 3000 dilution) (Santa Cruz) as primary antibodies followed by horseradish peroxidase (HRP)-conjugated goat anti-mouse (1:3000 dilution; Jackson ImmunoResearch, West Grove, PA, USA) as the secondary antibody. The detection was development using an enhanced chemiluminescence (ECL) (Thermo Scientific Waltham, MA, USA) system for WB and documented with ImageLab v6.1 in ChemiDoc MP system (BioRad).

### 2.7. Immunolocalization of T. vaginalis TvRAD51 in Zinc (Zn^2+^) and Cadmium (Cd^2+^)

For the immunolocalization of *T. vaginalis* TvRAD51, indirect immunofluorescence assays were conducted on parasites from the *T. vaginalis* CNCD147 isolate. The parasites were grown on sterile coverslips, both with and without Cd^2+^ (0.1 mM) or Zn^2+^ (1.6 mM). After cultivation, the parasites were washed with filtered 1x PBS (pH 7.0) and fixed in 4% paraformaldehyde for 1 h at 37 °C. The fixed trichomonads were then permeabilized using 0.2% Triton X-100 for 15 min at 25 °C, followed by two washes with filtered 1× PBS (pH 7.0). Blocking was performed with 0.2 M glycine for 1 h at 37 °C and 0.2% fetal bovine serum for 15 min at 25 °C. The slides were then incubated overnight at 4 °C with a mouse anti-human RAD51 (α-hRAD51) monoclonal antibody (diluted 1:100). After three washes with 1× PBS (pH 7.0), the slides were incubated for 1 h at 25 °C with fluorescein isothiocyanate (FITC)-conjugated goat anti-mouse immunoglobulin (1:200 dilution) (Jackson), followed by three washes with 1× PBS (pH 7.0). Finally, the slides were mounted using Vectashield-DAPI solution to stain nucleic acids and observed using a confocal microscope (Leica Inc., WetzlaR, Germany) (Supplementary Figure S2).

### 2.8. Statistical Analysis

The differences in mRNA *tvrad51* expression between the cation-exposed parasites (+UV, Zn^2+^, and Cd^2+^) and the control (CTRL) group were evaluated using an ANOVA, followed by a post hoc Tukey test based on the adjusted pixel intensity of bands of each experimental condition, compared with the control condition, and normalized with the intensity of bands of tubulin of three independent biological assays. This test was performed in ImageLab software (v6.1) with the densitometry analysis function (Bio-Rad). Statistical significance is denoted by asterisks in the figures, and the corresponding values are provided in the figure legends. All statistical analyses were conducted using GraphPad software (v9.3.0) (San Diego, CA, USA).

## 3. Results

### 3.1. In Silico Analysis of tvrad51 Nucleotide and TvRAD51 Amino Acid Sequences and Identification of Potential Relative Genes Involved in HR Machinery in T. vaginalis

**Figure 1:**
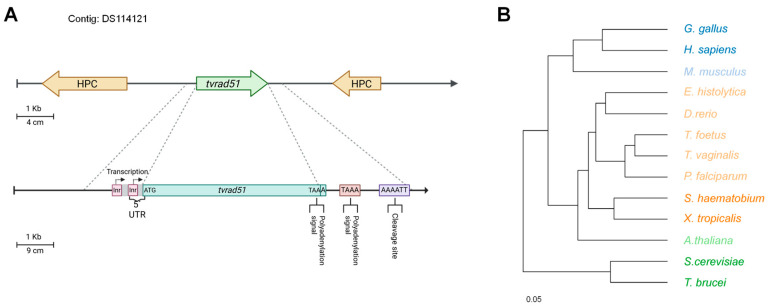
Identification of TvRAD51 gene in *T. vaginalis*. (**A**) Characterization of *tvrad51* genomic sequence. The 5′ and 3′ UTRs predicted from the *T. vaginalis* genome sequence TVAG_204070, the 5′ UTR contains two putative initiator promoter elements (Inr) (curved arrow and green boxes). Initiation (ATG) and stop codons (TGA) are indicated. Bar represents 1000 bp. (**B**) Phylogenetic analysis of TvRAD51. Number indicates bootstrap proportions (1000 replicates). Accession numbers: *Tritrichomonas foetus* (OHT06668.1), *H. sapiens* (NP_002866.2), *Arabidopsis thaliana* (NP_188928.2, *Schistosoma haematobium* (XP_012795174.1), *Gallus gallus* (NP_990504.1), *Xenopus tropicalis*: (NP_001016393.1), *Trypanosoma brucei* (AAD51713.1), *Plasmodium falciparum* (HB3 KOB62650.1), *E. histolytica* (XP_654076.1), *Saccharomyces cerevisiae* (EGA79221.1), *Danio rerio* (NP_998371.2), *Mus musculus* (NP_035364.1), and *T. vaginalis* (TVAG_204070).

To determine the possible expression of genes and proteins involved in the HR machinery, we focused on the in silico characterization and expression of the key recombinase TvRAD51. A putative sequence, annotated as “recombinase RAD51 (recA homolog)”, named *tvrad51* with an ORF of 990 bp located in the contig DS114121, was found in TrichDB with the access number TVAG_204070 (Gene Bank Accession: 4747955; UNIPROT: A2FXT7), which encodes a protein of 329 amino acids with a theoretical molecular weight of 35.5 kDa and a pI of 5.07 in the position 9677–10666 (+); it is flanked by conserved hypothetical proteins in the 5′ and 3′ UTRs, respectively (access numbers TVAG_204060 and TVAG_204090), (Figure 1A). The *tvrad51* gene sequence showed two putative Inr promoter elements at the 5′ UTR 9 and 23 nt upstream of the ATG initiation codon [23] (Figure 1A). No additional regulatory motifs upstream (M1, M2, M3, M4) 200 bp of the ATG initiation codon were found, as reported by [24] (Figure 1A). In the 3′ UTR, two predicted polyadenylation signal (PS) UAAA and processing sequences were also found. The first PS was included in the stop codon, and the others were found 20 nt downstream of the TAA stop codon. Additionally, one putative cleavage sequence (CS) was found 56 nt downstream of the first PS (Figure 1A). Three U-rich regions required for polyadenylation were also found downstream from the first putative cleavage site [25].

The *tvrad51* gene sequence exhibited two potential Inr promoter elements in the 5′ UTR, located nine and twenty-three nucleotides upstream of the ATG initiation codon [24,26] (Figure 1A). No typical Inr sequence or additional regulatory motifs in the upstream region within 200 bp of the ATG initiation codon were found, as reported by Smith et al., 2011 [24]. The phylogenetic analysis also showed that the evolutive history of TvRAD51 was more related to protozoan recombinases like PfRAD51, but in a separate branch together with *T. foetus* (Figure 1B).

According to the search of homologous putative genes involved in HR machinery, we found ten genes in the *T. vaginalis* G3 genome corresponding to RAD52 epistasis group recombinase, TvRAD51 (TVAG_204070), their paralogues TvRAD50 (TVAG_332660), TvRAD51D (TVAG_426330), and co-factor TvRad54B (TV_441050), the helicase TvBLM (TVAG_TVAG_255850), the nuclease TvMRE11 (TVAG_098295), TvXRCC2 (TVAG_155030), TvXRCC3 (TVAG_144570), the replication factor TvRPA (TVAG_294830), and the RAD51 filament formation protein TvBRCA2 (TVAG_473090) sequences, also with reported ESTs showing the expression of these genes under several grown conditions. Interestingly, TvRAD51 and TvRad54B are expressed in the G2/M cell cycle phase, suggesting their potential importance during mitotic events in *T. vaginalis* [27] (Table 1). A further search of TvRAD51 in the TrichDB database also showed four possible homologous gene sequences related to HsRAD51 as *rad51*pseudogene, *tvrad51*, *dmc1-1* and *-2*, and *xrcc3* (Table 2).

**Figure 2:**
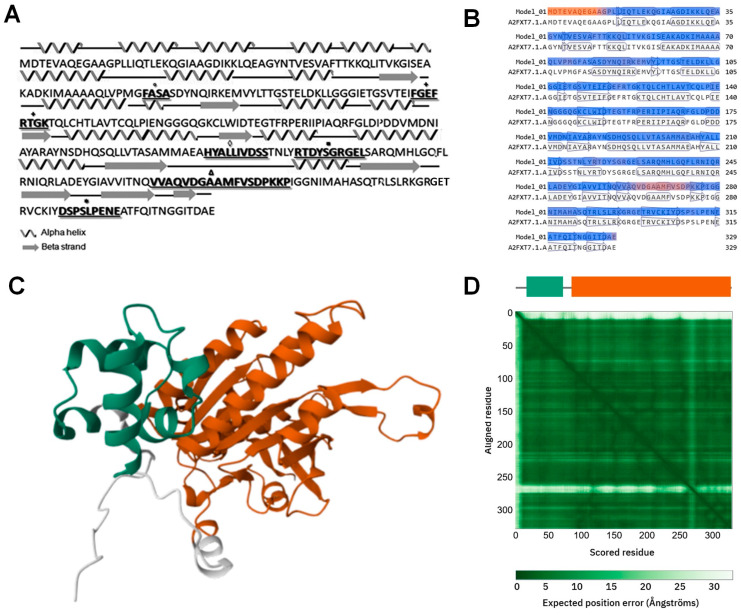
Prediction of secondary and tertiary structures of TvRAD51 in *T. vaginalis*. (**A**) Functional domains in TvRAD51 are shown in bold letters in the secondary structure, α-helix strands are represented as ribbons and β-strands as arrows; polymerization motif (PM) (·), Walker A (+) and B (◊) motif, L1 (**▪**) and L2 (**Δ**) regions, and ATP cap (*). (**B**) Model-Template alignment theoretical AlphaFold DB model of A2FXT7_TRIV3 [gene: A2FXT7_TRIV3 from *T. vaginalis* (strain ATCC PRA-98/G3)] with 100.00% Coverage. Analysis was performed in https://swissmodel.expasy.org/interactive/Y65n8C/models/ (accessed on 15 March 2025). (**C**) Three-dimensional model structure of TvRAD51 generated on Alphafold https://alphafold.ebi.ac.uk/entry/A2FXT7 (accessed on 15 March 2025) showing the two main domains (domain 1 in green and domain 2 in orange) according to the Encyclopedia of Domains (TED) with an average per-residue model confidence score (pLDDT) of 92.94. (**D**) Structural evaluation of the TvRAD51 model. The predicted aligned error (PAE) map indicates overall high confidence in the structure, with low uncertainty between the defined domains: domain 1 (residues 18–72, CATH 1.10.150.20) and domain 2 (residues 86–328, CATH 3.40.50.300). A localized region (~residues 250–300) exhibits increased structural variability.

The amino acid (aa) sequence of TvRAD51 was revealed, with the functional and structural conserved motifs of RAD51 (Figure 2A). TvRAD51 has a variant in the putative polymerization (PM) motif in the 77–80 aa residues, with the sequence FASA instead of FTTA of humans [28]. The ATPase Walker A (P-loop) motif in the 118–125 aa residues was revealed, with the conserved sequence FGEFRTGK. The Walker B motif of TvRAD51 in the 206–216 aa residues forming the ‘RecA fold’, the conserved core of RAD51/RecA/RadA-like factors in eukaryotes, and the single-stranded DNA (ssDNA) binding loops L1 in the 221–230 aa residues, were similar to HsRad51. The L2 (260–277 aa residues) were the most divergent sequence, and the ATP cap (309–317 aa residues) was also conserved at the C-terminal (Figure 2A). These last residues are essential for nucleofilament assembly and ATP hydrolysis in RAD51 recombinases [28]. Secondary and three-dimensional structures were predicted for TvRAD51, showing sixteen α-helix and nine β-strands, in a similar arrangement to *H. sapiens* RAD51 (pdb: 5JZC) (Figure 2B). This arrangement might suggest the functionality of TvRAD51 and would be explained due to the fact that HsRAD51 is structurally similar and performs the dynamic nucleoprotein filament on ssDNA [27,28] (Figure 2C,D).

**Figure 3:**
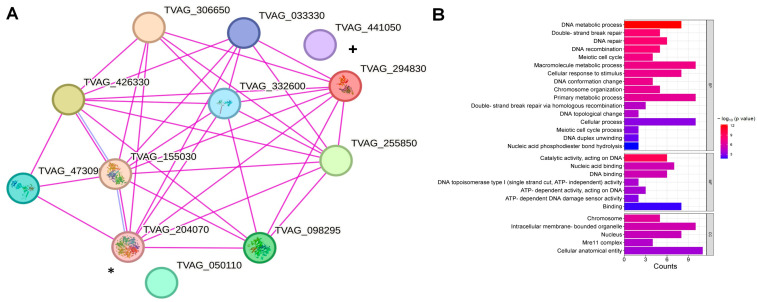
Core protein–protein interaction network analysis of putative HR machinery proteins in *T. vaginalis*. (**A**) The network depicts interactions among 12 proteins, where colored nodes represent query proteins and their first shell of interactors, while white nodes indicate the second shell of interactors. Empty nodes correspond to proteins with unknown 3D structures, and filled nodes denote proteins with known or predicted 3D structures. Edges shown in magenta represent experimentally validated interactions. TvRAD51 [TVAG_204070 (A2FXT7) is marked with an asterisk (*) and TvRAD54B [TVAG_441050 (A2FSS0) is marked with a plus symbol (+)],. The full list of node names and accession numbers and other analyzed parameters are provided in Supplementary Figure S3. (**B**) GO enrichment analysis illustrating biological processes, molecular functions, and cellular components associated with TvRAD51 and its interacting proteins. Statistical significance is expressed as −log10(*p*-value), with gene counts shown for each category.

To corroborate the possible interaction of the homologous HR proteins identified by ESTs, we performed a protein interaction cluster to obtain the possible interaction nodes of TvRAD51. According to the protein–protein interaction analysis, TvRAD51 (A2FXT7) can directly interact with several proteins of the DNA binding/repair of *T. vaginalis* as a ATP-dependent DNA helicase, the RecQ family protein (1447 aa) that presents a BLM domain (A2DYY2, TvBLM), a putative serine/threonine protein phosphatase (562 aa) with a TvMRE11 domain (A2ECB0), an uncharacterized protein (590 aa) but with the Rep-A_N domian (TvRPA, A2DL41), a zinc-hook domain-containing protein (1292 aa) similar to TvRAD50 (A2FAD3), and a BRCA2 repeat family protein (1664 aa) TvBRCA2 (A2ERV5). Parologous RAD51, an SNF2-family N-terminal-domain-containing protein (1107 aa), TvRAD54B (A2FSS0), does not show a direct interaction with TvRAD51. Other proteins potentially involved in the machinery were found, A2DYQ0, corresponding to the RECA_2 domain (288 aa), and A2G1B8, also with the RECA_2 domain (328 aa), respectively. Other predicted physical partners were found, such as TVAG_050110, an uncharacterized protein but with a Mei5 domain (score: 0.540) critical for critical for HR repair, TVAG_155030, a putative meiotic recombination protein DMC1/LIM15 homolog, belonging to the RecA family (0.976), type IA topoisomerases TVAG_306650 and TVAG_033330 (both proteins score 0.810), and TVAG_332540, a zinc-hook domain-containing protein with a RAD50 domain (Score: 0.956) (Figure 3A). The Gene Ontology (GO) enrichment analysis showed a significant association with biological processes such as DNA metabolic processes, DSB break repair, and DNA recombination, and metabolic functions such as catalytic activity and ATP-dependent DNA damage sensing and acting activity, mainly in the MRE11 complex, a multi-subunit nuclease that is composed with RAD50 and Nbs1/Xrs2, and is involved in checkpoint signaling and DNA replication, mainly in nucleus with highly significant FDR values (Figure 3B). These results suggest that TvRAD51 plays a key role in maintaining genome stability, which may be relevant for the biology and adaptation of *T. vaginalis*, and also appears to be strongly involved in DNA binding and repair mechanisms through HR. Its interaction network suggests a connection with other key proteins involved in DNA repair and meiotic processes. These findings support the conserved role of RAD51 in DNA repair and genome stability maintenance, which could be relevant for the biology and virulence of *T. vaginalis*; however, the protein interactions depend on local conditions, so only a subset of database-reported PPIs are active in a given experiment. Still, such databases are widely used as gold standards, assuming these interactions are relevant under the study’s conditions [29].

### 3.2. Expression of TvRAD51 in Trichomonas vaginalis in Response to UV Insult

**Figure 4:**
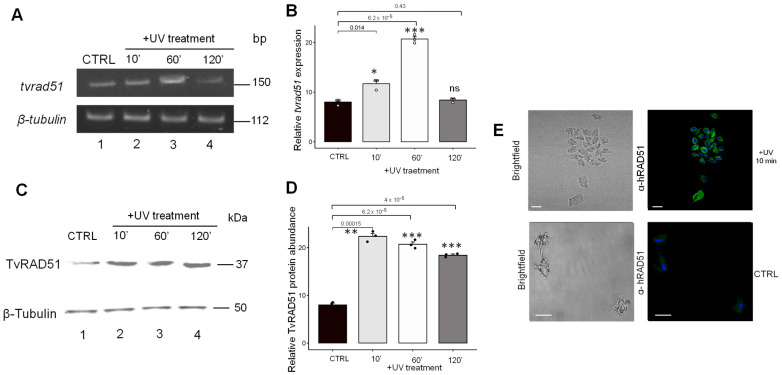
Expression and abundance of the TvRAD51 protein in *T. vaginalis* following UV-irradiation treatment (**A**). Expression of *tvrad51* by RT-PCR assays with specific primers for *tvrad51* fragment gene (150 bp) showing gene expression upregulated by irradiation (lane 1: control (CTRL), lane 2: 10 min, lane 3: 30 min, lane 4: 60 min, and lane 5: 120 min). (**B**) Densitometric analysis of pixel intensity for *tvrad51* expression. (**C**) 37 kDa band immunorecognized by anti-hRAD51 antibodies (lane 1: control (CTRL), lane 2: 10 min, lane 3: 60 min, and lane 4: 120 min). (**D**) Densitometric analysis of pixel intensity for TvRAD51 protein abundance of three biological independent assays were performed and statistical significance was determined by ANOVA and post hoc Tukey test, comparing the standard deviations (SDs) of each condition. Relative gene expression and protein abundance was normalized with protein loading control: anti-human α-tubulin (50 kDa) or β-tubulin gene fragment (112 bp). A *p*-value of <0.001 was considered statistically significant (***), *p* < 0.01 (**), *p* < 0.05 (*), ns: not statistically significance. (**E**) Localization of TvRAD51 in 10 min of UV-treated parasites (upper panel) and CTRL (lower panel) of *T. vaginalis* CNCD 147 incubated with α-hTvRAD51 followed by FITC-conjugated goat anti-mouse IgG. Weak green signal (FITC) was detected in CTRL condition. The slides were observed at 40× magnification by confocal microscopy (Leica). Scale bar indicates 60 μm.

To evaluate the expression of the recombination machinery native protein RAD51 in this protozoan parasite, we determine the TvRAD51 expression in *T. vaginalis*; after a ultraviolet light (UV) genotoxic insult inductor, we performed RT-PCR and Western blot and immunofluorescence assays using a monoclonal α-human RAD51 antibody from (+UV) irradiated and non-irradiated parasites. RT-PCR assays were performed to determine the *tvrad51* gene expression, and a higher intensity of bands was found in irradiated parasites after 60 min of UV (2.5-fold increase) (Figure 4A, higher panel lane 2) compared with parasites without irradiation and the other conditions, including time periods of 10 min (1.4-fold increase) and 120 min (no changes observed) (Figure 4A, higher panel lanes 3–4), and a 112 bp region of the β-tubulin gene fragment was used as the control (Figure 2A, lower panel lanes 1–4), according to densitometric analysis (Figure 4B). To determine the protein abundance, using the WB assay, an increased band of 37 kDa (expected size) in +UV irradiation-treated parasites at 10 min was found, which corresponds to endogenous TvRAD51 (Figure 4C, higher panel lane 2). These results showed that according to densitometric analysis, in parasites with +UV irradiation, TvRAD51 has a 2.8-fold increase in response to 10 min, 2.6-fold-increase in response to 60 min, and 2.4-fold increase in response to 120 min compared to the untreated control, but in a short time period compared with the gene expression (Figure 4D). To confirm the most abundant protein in a short time period, the immunofluorescence assay was performed at 10 min, the time with the highest abundance of TvRAD5, and focal structures were observed (Figure 4E).

### 3.3. The Expression, Protein Abundance, and Localization of the TvRAD51 Protein Is Modulated by Zinc Exposure in T. vaginalis

**Figure 5:**
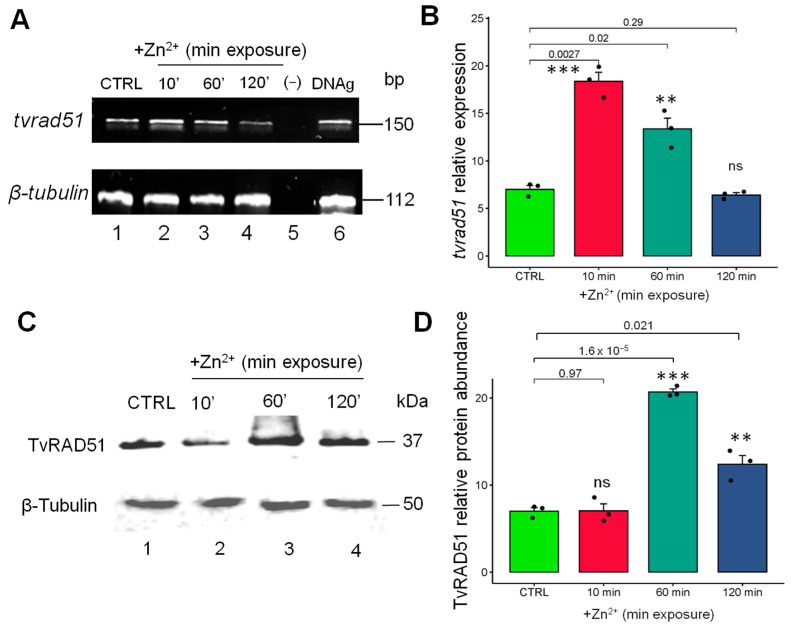
Expression and abundance of the TvRAD51 protein in *T. vaginalis* following Zn^2+^ treatment (1.6 mM). (**A**) Expression of *tvrad51 by* RT-PCR assays with specific primers for *tvrad51* fragment gene (150 bp), showing the time course gene expression upregulated by Zn^2+^1.6 mM (lane 1: control (CTRL), lane 2: 10 min, lane 3: 30 min, lane 4: 60 min, and lane 5: 120 min). (**B**) Densitometric analysis of pixel intensity for *tvrad51* expression. (**C**) 37 kDa band immunorecognized by anti-hRAD51 antibodies (lane 1: control (CTRL), lane 2: 10 min, lane 3: 60 min, lane 4: 120 min, and lane 5: 24 h). (**D**) Densitometric analysis of pixel intensity for TvRAD51 protein abundance of three biological independent assays were performed and statistical significance was determined by ANOVA and post hoc Tukey test, comparing the standard deviations (SDs) of each condition. Relative gene expression and protein abundance was normalized with protein loading control: anti-human α-tubulin (50 kDa) or β-tubulin gene fragment (112 bp). A *p*-value of <0.001 was considered statistically significant (***), *p* < 0.05 (**), ns: not statistically significance.

The mechanisms underlying *tvrad51* basal gene expression and the effect of cations are unknown. To elucidate this, RT-PCR assays were performed to examine *tvrad51* gene expression quantitatively, and according to the densitometric analysis (Figure 5B), the results showed that the recombinase mRNA levels increased by 3.1-fold in parasites grown with Zn^2+^ at 10 min of exposure (Figure 5A line 2) and by 1-fold at 60 min of exposure (Figure 5A line 3), whereas no changes were observed upon 120 min of exposure (Figure 5A line 4) compared with parasites grown without Zn^2+^ conditions (CTRL) (Figure 5A line 1). The immunodetection of the TvRAD51 protein in *T. vaginalis* was performed using extracts from parasites treated with 1.6 mM Zn^2+^ for different times of exposure (10 min, 60 min, 120 min, and 24 h), as well as a control (CTRL) without cation treatment. The protein immunodetection was carried out using the mouse anti-human RAD51 monoclonal antibody and an expected molecular weight of ~37 kDa was recognized, corresponding to the predicted RAD51 molecular weight observed in the CTRL condition, demonstrating the basal expression of this recombinase in the parasite (Figure 5C, line 1). However, according to densitometric analysis (Figure 5D), the protein abundance of TvRAD51 showed a non-significant abundance increase 10 min after parasite recovery (Figure 5C line 2) and a 2.8-fold increase at 60 min of Zn^2+^ treatment, which was the time point with the highest abundance (Figure 5C, lane 3). The abundance levels of TvRAD51 subsequently decreased at 120 min, with a 1.5-fold increase (Figure 5C, lane 4) compared to the CTRL protein abundance (Figure 5C, lane 1).

**Figure 6:**
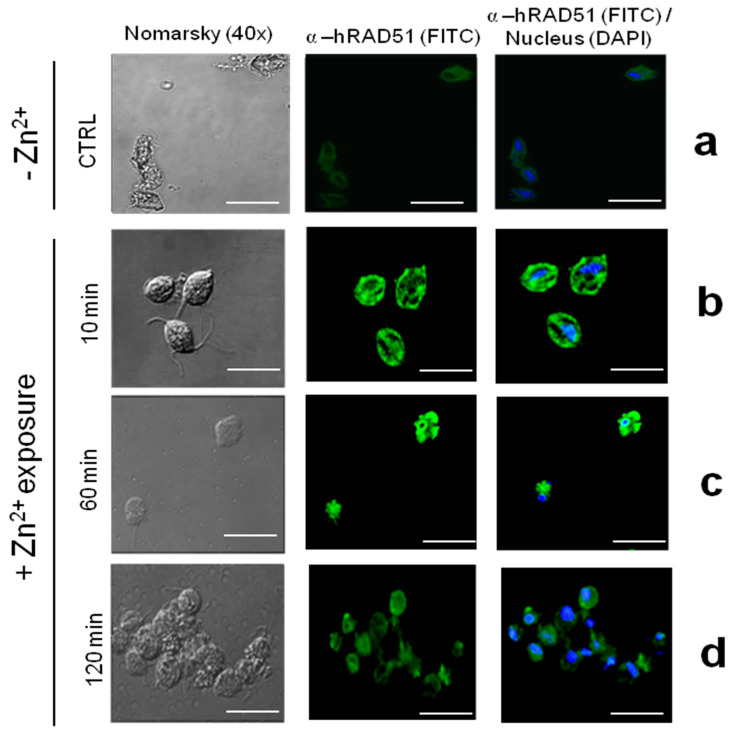
Localization of TvRAD51 in *T. vaginalis* CNCD 147 isolate in Cd^2+^ exposure. (**a**) Parasites grown with no added Zn^2+^ in medium (-Zn^2+^, CTRL), fixedm and incubated with α-hTvRAD51, followed by FITC-conjugated goat anti-mouse IgG and DAPI for nucleus stain. (**b**) Localization of TvRAD51 in parasites exposed to zinc (+Zn^2+^) for 10 min, (**c**) 60 min, and (**d**) 120 min of exposure. Green signal (FITC) was merged with DAPI (blue signal). Two independent assays were performed, and the slides were observed at 40× magnification by confocal microscopy (Leica). Scale bar indicates 60 μm.

To determine the localization of the TvRAD51 protein within the *T. vaginalis* parasite after the Zn^2+^ treatment (1.6 mM) at time points of 10, 60, and 120 min, an indirect immunofluorescence assay was performed on slides with fixed and permeabilized parasites (Figure 6). The parasites were incubated with the monoclonal anti-hRAD51 antibody and the confocal microscopy showed that the protein exhibited a discrete signal localized in the cytoplasm of parasites (Figure 6a), with an increase in the fluorescence intensity of the protein in parasites treated with Zn^2+^ at 10 and 60 min (Figure 6b,c), but a decrease at 120 min of recovery (Figure 6d). This suggests the involvement of the protein in response to a short insult duration, indicating potential DNA repair mechanisms and adaptation to environmental stress conditions in the parasite due to a not apparent signal being recognized in the CTRL condition (Figure 6a).

### 3.4. The Expression and Abundance of TvRAD51 Is Also Modulated by Cadmium in T. vaginalis but at an Early Time Point Compared to Zinc

**Figure 7:**
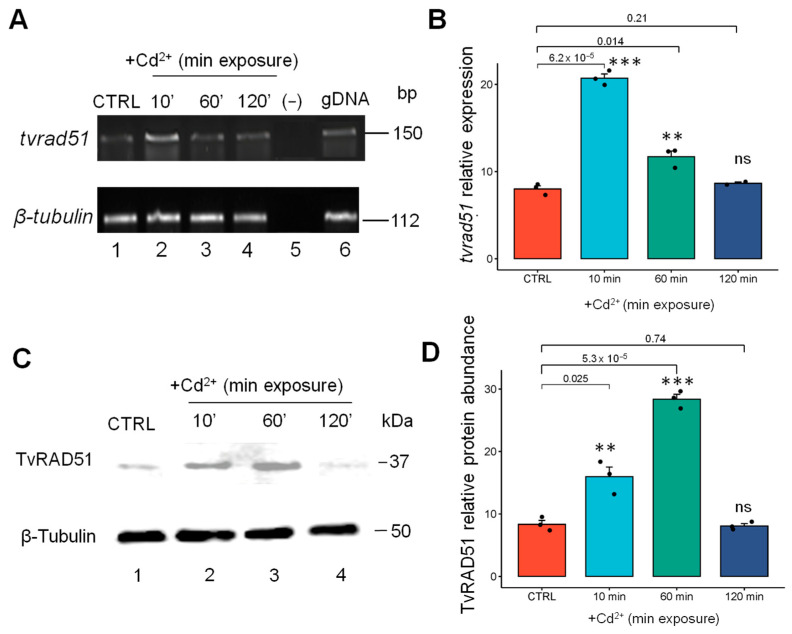
Expression and protein abundance of the TvRAD51 protein in *T. vaginalis* following Cd^2+^ treatment (0.1 mM). (**A**) Expression of *tvrad51 by* RT-PCR assays with specific primers for *tvrad51* gene, showing the time course gene expression upregulated by Zn^2+^1.6 mM (lane 1: control (CTRL), lane 2: 10 min, lane 3: 30 min, lane 4: 60 min, and lane 5: 120 min). (**B**) Densitometric analysis of pixel intensity for *tvrad51* expression. (**C**) 37 kDa band immunorecognized by anti-hRAD51 antibodies (lane 1: control (CTRL), lane 2: 10 min, lane 3: 60 min, lane 4: 120 min, and lane 5: 24 h). (**D**) Densitometric analysis of pixel intensity for TvRAD51 protein abundance. Two independent assays were performed in technical triplicate, and statistical significance was determined by ANOVA and post hoc Tukey test, comparing the standard deviations (SDs) of each condition. Relative gene expression and protein abundance was normalized with protein loading control: anti-human α-tubulin (50 kDa) or β-tubulin gene fragment (112 bp). A *p*-value of <0.05 was considered statistically significant (***), *p* < 0.05 (**), ns: not statistically significance.

The *tvrad51* gene expression was also quantitatively evaluated in the Cd^2+^ condition; according to the densitometric analysis (Figure 5B), the results showed that gene expression increased by 2.3-fold in parasites exposed for 10 min (Figure 7A, line 2), by 1.2-fold after 60 min (Figure 7A line 3), and no changes were observed upon 120 min of exposure (Figure 7A line 4) compared with CTRL parasites.

The immunodetection of TvRAD51 was also conducted using extracts from parasites treated with 0.1 mM Cd^2+^ for the same Zn^2+^ exposure times (10 min, 60 min, 120 min, and 24 h), along with a CTRL. The parasite recombinase ~37 kDa band was detected using the same mouse anti-human RAD51 monoclonal antibodies, and a band with a molecular weight of 37 kDa was significantly increased at 10 min (2.2 fold increase) (Figure 7A, lane 2), but slightly decreased at 60 min (1.5 fold increase) (Figure 7A, lane 3), and at 120 min, no changes in increase were observed (Figure 7A lane 4) in the Cd^2+^ treatment compared to the CTRL (Figure 7A, lane 1), according to the densitometric analysis (Figure 7B). According to the densitometric analysis of band intensity, TvRAD51 protein abundance then decreased at 120 min (Figure 7A, lane 5) and remained low at 24 h, indicating that at 10 min, there was a 0.8-fold higher relative expression compared to the control. This time point exhibited the highest abundance of TvRAD51, and these results were statistically significant (*p* < 0.001) (Figure 7B).

**Figure 8:**
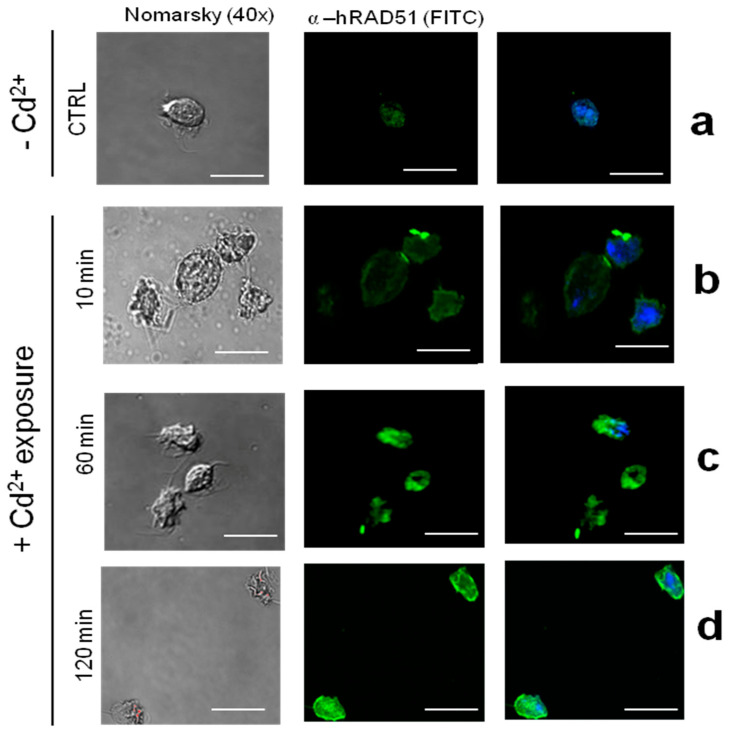
Localization of TvRAD51 in *T. vaginalis* CNCD 147 isolate during Cd^2+^ exposure. (**a**) Parasites grown with no added Cd^2+^ in medium (-Cd^2+^, CTRL), fixed, and incubated with α-hTvRAD51, followed by FITC-conjugated goat anti-mouse IgG and DAPI for nucleus stain. (**b**) Localization of TvRAD51 in parasites exposed to zinc (+Cd^2+^) 10 min, (**c**) 60 min, and (**d**) 120 min of exposure. Green signal (FITC) was merged with DAPI (blue signal). Two independent assays were performed, and the slides were observed at 40× magnification by confocal microscopy (Leica). Scale bar indicates 60 μm.

Immunofluorescence and confocal microscopy revealed that TvRAD51 had a modest localization in the cytoplasm in the parasite under the control condition (CTRL) (Figure 8a). The recombinase showed an increase in the fluorescence intensity of the protein in parasites treated with Cd^2+^ for 10 min, with a discrete signal in nucleus (Figure 8b), while the intensity was decreased at 60 and 120 min of treatment (Figure 8c,d). The TvRAD51 protein was most prominently observed at 10 min, suggesting that the parasite adapts to Cd^2+^ levels differently than to Zn^2+^ levels. This result is partially consistent with TvRAD51 recognition in parasite extracts, where this recombinase is detected more after 60 min, but the expression begins within 10 min of exposure. These results suggest that the adaptation of the parasite to Cd^2+^ might not be effective, as this metal is possibly not typical of the urogenital tract, and this also suggests that the parasite may have DNA repair mechanisms and can adapt to stress conditions, such as exposure to other toxic non-typical metals like Cd^2+^.

The immunofluorescence and confocal microscopy revealed that TvRAD51 had a modest localization in the cytoplasm in the parasite under the control condition (CTRL) (Figure 8a). The recombinase showed an increase in the fluorescence intensity of the protein in parasites treated with Cd^2+^ for 10 min, with a discrete signal in the nucleus (Figure 8b), while the intensity was decreased at 60 and 120 min of treatment (Figure 8c,d). The TvRAD51 protein was most prominently observed at 10 min, suggesting that the parasite adapts to Cd^2+^ levels differently than to Zn^2+^. This result is partially consistent with TvRAD51 recognition in parasite extracts, where this recombinase is detected more after 60 min, but the expression begins within 10 min of exposure. These results suggest that the adaptation of parasite to Cd^2+^ might not be effective, as this metal is possibly not typical of the urogenital tract, and this also suggests that the parasite may have DNA repair mechanisms and can adapt to stress conditions, such as exposure to other toxic non-typical metals like Cd^2+^.

## 4. Discussion

### 4.1. HR in Protozoa and Their Impact

In eukaryotes, HR is a crucial mechanism that not only preserves genome integrity, but also supports genomic versatility and plasticity [30]. In many pathogenic organisms, the recombination events may serve as a strategy to survive immune pressure from the host and adapt to environmental factors [14]. The characterization of recombination events, as well as the expression and involvement of the recombinase protein RAD51, have been extensively studied in the context of two-ended DSB repair in various parasitic organisms, mainly in protozoan parasites [15]. Although *T. vaginalis* is considered an asexual unicellular organism, the presence of six condensed chromosomes [26] and multiple meiotic genes—such as *tvrad51* and *DMC1*—and the annotation of a putative histone H2AX in *T. vaginalis* (TVAG_447860) with multiple phosphorylation sites suggests the existence of a HR mechanism. Similar to other protozoan parasites like *Entamoeba histolytica* [26], this mechanism may contribute to the maintenance of genome integrity or the antigenic variation of surface proteins, such as TvBspA-like molecules [30,31,32,33]. Additionally, the highly repetitive genome of *T. vaginalis* [34] may support drug resistance, further reinforcing the notion of functional HR in this parasite [35,36,37,38,39].

### 4.2. T. vaginalis and TvRAD51 Network

Our in silico analysis confirmed the conserved structure of TvRAD51 and its predicted interactions with HR-related proteins, supported by a STRING network analysis showing strong protein–protein interactions [16]. These interactions implicate TvRAD51 as part of a coordinated repair complex that is potentially involved in genomic adaptation and stability (Figure 3A). The molecular function analysis further supports the possible role of these proteins in DNA repair processes, as nucleases and DNA-binding functions were identified, which may be associated with DNA repair, genetic editing, or the degradation of damaged genetic material (Figure 3B). Although further validation is required to determine the exact pathways in which this protein group is involved, these findings provide valuable insights into their potential functions. Similarly, the functional enrichment analysis suggests that these proteins may be localized in the nucleus, forming protein complexes. The DNA damage can be induced by genotoxic agents (as drugs) in *T. vaginalis*, where a resection step is performed by MRN-like complex mediated by TvMRE11 and TvRAD50.

### 4.3. TvRAD51 Gene and Protein Expression Under Genotoxic Conditions

In this work, we identified native recombinase TvRAD51 in the parasite. RT-qPCR and Western blot analyses revealed a significant upregulation of *tvrad51* expression and protein accumulation upon UV (Figure 4), Zn^2+^ (1.6 mM), and Cd^2+^ (0.1 mM) exposure (Figure 5 and Figure 6). Notably, TvRAD51 expression peaked at early time points following metal exposure—particularly at 10 and 60 min—before declining at 120 min and 24 h. This dynamic expression pattern suggests that TvRAD51 is promptly mobilized as part of a rapid genotoxic response, possibly regulated by early signaling cascadesm triggered by oxidative or structural DNA stress or replication conflicts [40,41]. When the parasite is exposed to various endogenous treatments, such as the genotoxic agents presented in this study, or due to erroneous replications caused by stress, such as a change in medium or an increase in toxic agents for the organism [41,42], the RAD51 protein primarily localizes to nuclear foci to repair genetic lesions [43]. Under the Zn^2+^ treatment at a concentration of 1.6 mM in *T. vaginalis*, we observed a significant increase in TvRAD51 proteins in the nucleus after 10 and 60 min of treatment, compared to a control (CTRL) or after 120 min under the same condition (Figure 6). This was observed through the fluorescence of the protein localized in the parasite’s nucleus. In contrast, under the Cd^2+^ treatment at a concentration of 0.1 mM, an increase in nuclear TvRAD51 proteins was only observed after 60 min, with no changes detected between the control, 10 min, and 120 min treatments under the same condition (Figure 7).

### 4.4. Differential Responses to Zn^2+^ and Cd^2+^

The differential abundance observed with the specific concentrations and treatment times of Zn^2+^ and Cd^2+^ indicates that TvRAD51 is not constantly active in the parasite. The differential expression profiles observed between Zn^2+^ and Cd^2+^ exposures suggest that *T. vaginalis* may be more adapted to handle physiologically relevant metals like Zn^2+^, while Cd^2+^ triggers a transient and potentially less effective repair response. These findings highlight the relevance of TvRAD51 not only in maintaining genome stability but also as a potential target for disrupting DNA repair in the parasite under stress conditions. This may reflect the parasite’s evolutionary adaptation to Zn^2+^—a more physiologically relevant metal in the urogenital tract—contrasted with Cd^2+^, a xenobiotic metal not typically encountered in this niche. These findings align with studies indicating that Cd^2+^ induces DNA damage through oxidative stress and inhibits repair pathways in other eukaryotes [44,45]. In *T. vaginalis*, the inability to sustain TvRAD51 expression during prolonged Cd^2+^ exposure could point to a limited repair capacity or toxic interference with regulatory proteins due their exposition to the fluctuating conditions of the human genitourinary tract during infection, which could cause direct and indirect damage to the parasite’s DNA (mainly by DSBs), compromising its survival. The excess of Zn^2+^ and Cd^2+^ can also induce direct genotoxicity by binding to the nucleophilic groups of deoxyribose and the nitrogenous bases of the DNA molecule, leading to chemical modifications [46], and as a result, damaging the DNA molecule. Moreover, these cations can also indirectly damage genetic material by inducing the accumulation of reactive oxygen species (ROS) and free radicals [47]. Additionally, Cd^2+^ has been shown to replace Zn^2+^ in p53, inhibiting its DNA-binding activity and preventing cell cycle arrest following genetic damage [48]. Cd^2+^ is also capable of decreasing GSH levels and activating various signaling cascades sensitive to ROS accumulation, such as phospholipase C (PLC), protein kinase C (PKC), and mitogen-activated protein kinases (MAPKs) [49,50]. Given *T. vaginalis*’ exposure to fluctuating cation concentrations during infection, the activation of HR and TvRAD51 expression may represent an adaptive strategy to survive host-mediated stress, including inflammatory ROS and antimicrobial metals. The early and transient TvRAD51 response to Cd^2+^ also highlights a potential susceptibility to environmental toxicants. Understanding this response is relevant not only for basic parasitology but also for considering therapeutic approaches that might exploit the parasite’s limited DNA repair adaptability under metal-induced stress.

### 4.5. Subcellular Localization of TvRAD51 and Implications for DNA Repair

Immunofluorescence analyses revealed that TvRAD51 localizes to the nucleus shortly after metal exposure, consistent with its role in HR repair. The strongest nuclear localization was observed at 10 min post-treatment, particularly under Cd^2+^ exposure, followed by cytoplasmic redistribution at later time points [43,51] (Figure 8). This nuclear relocalization may indicate active engagement in DSB repair, followed by degradation or redistribution once repair is complete. These dynamics parallel RAD51 behavior in other eukaryotes, where post-repair downregulation is critical to avoid inappropriate recombination events [52].

### 4.6. Potential Biological and Clinical Implications

In certain time-specific treatments, the protein is not present, suggesting that TvRAD51 is activated in response to genetic damage only when necessary for the repair process in the parasite. It has been demonstrated that Cd^2+^ can regulate specific proteins in the parasite, such as the *tvmt*-1 gene, which encodes for metallothionein 1 (TvMT-1), and significantly induces its expression and the positive regulation of TvMT-1 in the cytoplasm of parasites grown in the presence of Cd^2+^ [19]. This is supported by studies showing that TvRAD51 is not always active [53]. The combination of genetics, molecular biology, biochemistry, and bioinformatics has allowed assigning specific roles to the necessary proteins upstream and downstream during HR and general recombination. This has led to a better understanding of the mechanisms at play in recombination. The principles of DNA pairing and invasion with the different processes that regulate it have also been established. However, many unanswered questions remain, especially in parasitic protists and their environmental relationship and DNA repair mechanisms, which rely on HR to survive, adapt, and vary their genome.

## 5. Conclusions

This study provides the first experimental evidence that Trichomonas vaginalis expresses the recombinase TvRAD51 in response to genotoxic stress caused by UV radiation and exposure to sublethal concentrations of Zn^2+^ and Cd^2+^. Our results demonstrate that TvRAD51 is transcriptionally and translationally upregulated under these conditions, showing rapid and dynamic expression patterns, especially at early time points. Furthermore, the nuclear localization of TvRAD51 shortly after exposure suggests its active role in HR-mediated DNA repair.

Altogether, this work expands our understanding of the DNA damage response in *T. vaginalis* and opens new perspectives on how this parasite may survive, adapt, and potentially develop resistance mechanisms in the challenging microenvironment of the male urogenital tract.

## Figures and Tables

**Table 1:** Homologous genes of RAD52 epistasis group found in *T. vaginalis* G3 genome and reported ESTs under different growth conditions.

Gene Name	ID (TrichDB)	EST ID	EST Length	Condition *
TvRAD51	TVAG_204070	TvG004A11	693	G2/M trophozoite (TvG2M)
TvXRCC3	TVAG_144570	LK995170	538	TvC
		TvC127B06	538	Cold-induced pseudocyst (TvCS) ^&^
TvBRCA2	TVAG_473090	JK970464	422	*T. vaginalis* log-phase trophozoite library (TvE)
		TvE051F07	422	Normal unsynchronized culture (TvEST)
TvRAD54B	TVAG_441050	LK991215	502	TvC
		TvC074A09	706	Cold-induced pseudocyst (TvCS) ^&^
		TvE077C08	461	Normal unsynchronized culture (TvEST)
TvRAD50	TVAG_332600	CV204056	365	Non-normalized T1 cDNA library ^#^
		CV204057	452	Non-normalized T1 cDNA library ^#^
TvMRE11	TVAG_098295	GT110554	700	Normalized cDNA library from *T. vaginalis* trophozoites grown in vitro (mid-log phase)
		GT110567	700	Normalized cDNA library from *T. vaginalis* trophozoites grown in vitro (mid-log phase)

* According to library. ^&^ TvCS: cold-induced pseudocyst conditions, in which *T. vaginalis* was exposed to 4 °C for 4 h to induce a transient pseudocystic morphology as model of stress responses. ^#^ Non-normalized T1 cDNA: a complementary DNA library generated without transcript normalization, derived from the T1 isolate under standard growth conditions.

**Table 2:** Identification of possible homologous sequences to TvRAD51 in *T. vaginalis*.

Gene ID	Gene	Protein Length (aa)	Uniprot ID	Identity % *
TVAG_021820	rad51pseudogene	115	A2DHG2	53.91%
TVAG_204070	*tvrad51*	329	A2FXT7	69.91%
TVAG_021810	*dmc1*	153	A2DHG1	79.08%
TVAG_155030	*dmc1*	338	A2FY08	53.15%
TVAG_144570	*xrcc3*	328	A2G1B8	23.68%

* Identity according to HsRAD51 amino acid sequence (gene ID: 5888).

## Data Availability

The original data presented in the study are openly available at TrichDB (http://trichdb.org/trichdb/ (accessed on 30 April 2025)) for reported ESTs and sequences, two and three-dimensional homology modeling of TvRAD51 based on *H. sapiens* template obtained from the Protein Data Bank (http://www.rscb.org/ (accessed on 5 November 2024)) (PDB: 5JZC). STRING analyses were performed in STRING https://version-12-0.string-db.org/ (accessed on 15 March 2025) with TVAG_204070 as query https://string-db.org/ (accessed on 15 March 2025).

## Supplementary Materials

The following supporting information can be downloaded at: https://www.mdpi.com/article/10.3390/pathogens14060565/s1, Figure S1: Original agarose gels of end-point PCR. Figure S2: Images of IFA images of *T. vaginalis* used in this work. Figure S3: Protein–protein interaction cored network analysis with putative proteins of *T. vaginalis* HR machinery.

## Abbreviations

The following abbreviations are used in this manuscript:

HR	Homologous recombination
UV	Ultraviolet light
DSB	Double-strand break
Zn^2+^	Zinc cation
Cd^2+^	Cadmium cation
CTRL	Control condition
