# Supplementary material for: Modulation of TvRAD51 Recombinase in Trichomonas vaginalis by Zinc and Cadmium as a Potential Mechanism for Genotoxic Stress Response
Source: Pathogens. 2025 Jun 5;14(6):565. doi: 10.3390/pathogens14060565 (PMC12195773; doi:10.3390/pathogens14060565)

**Figure S1.** Original agarose gels of end-point PCR.

RT-PCR Tv+UV B-tubulin assay 1

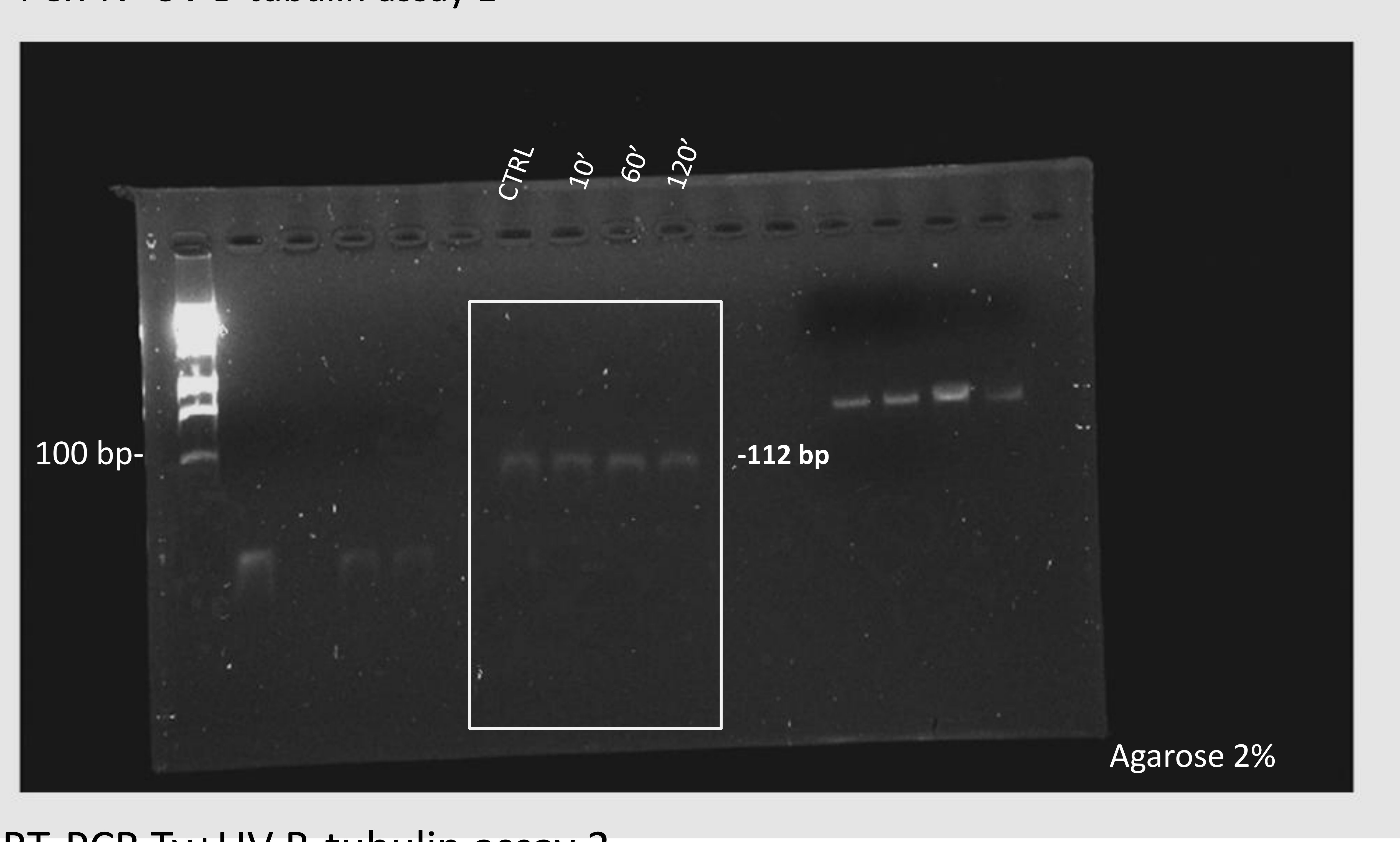


RT-PCR Tv+UV B-tubulin assay 2

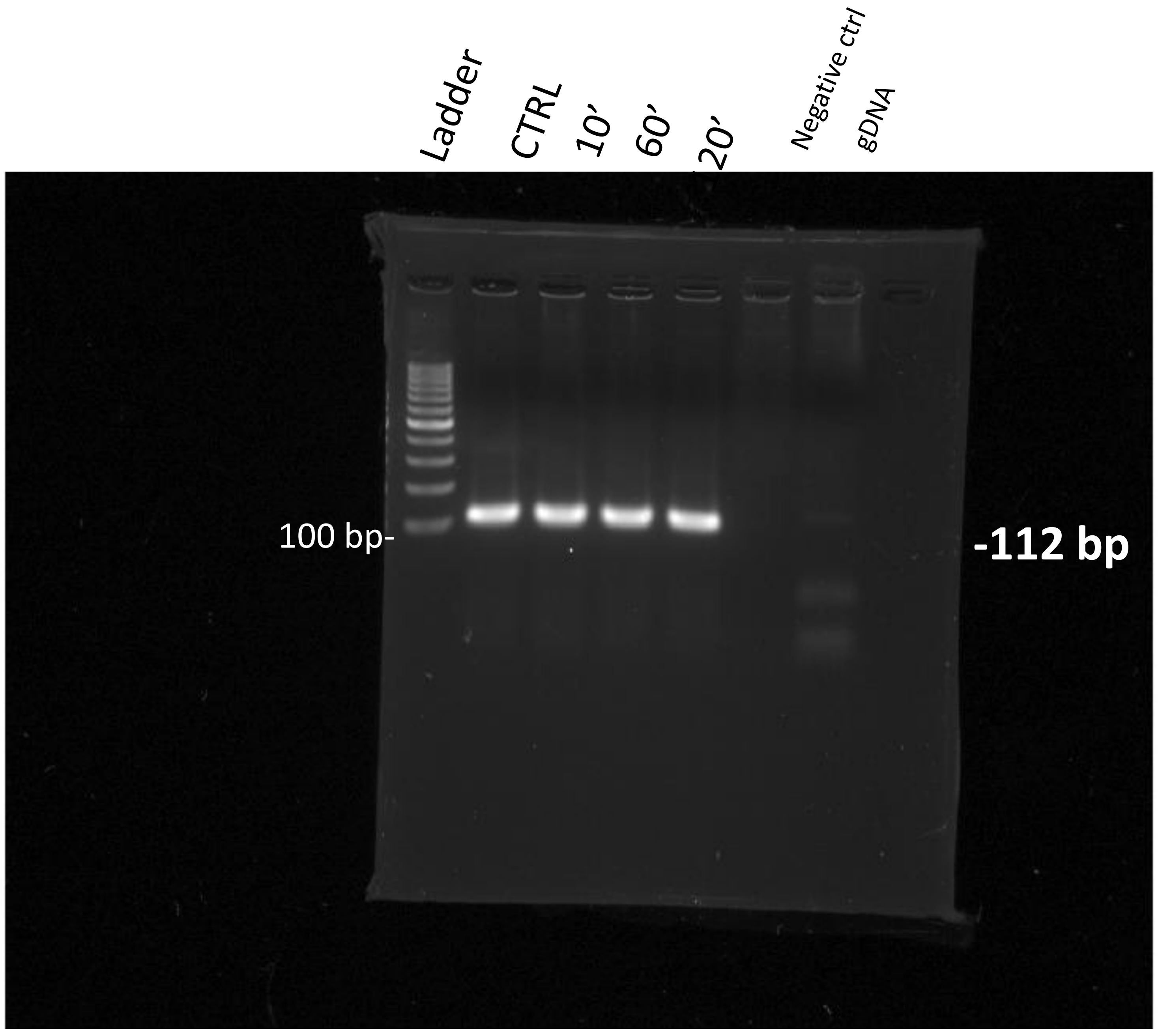


Agarose 2%

RT-PCR Tv+UV B-tubulin assay 3

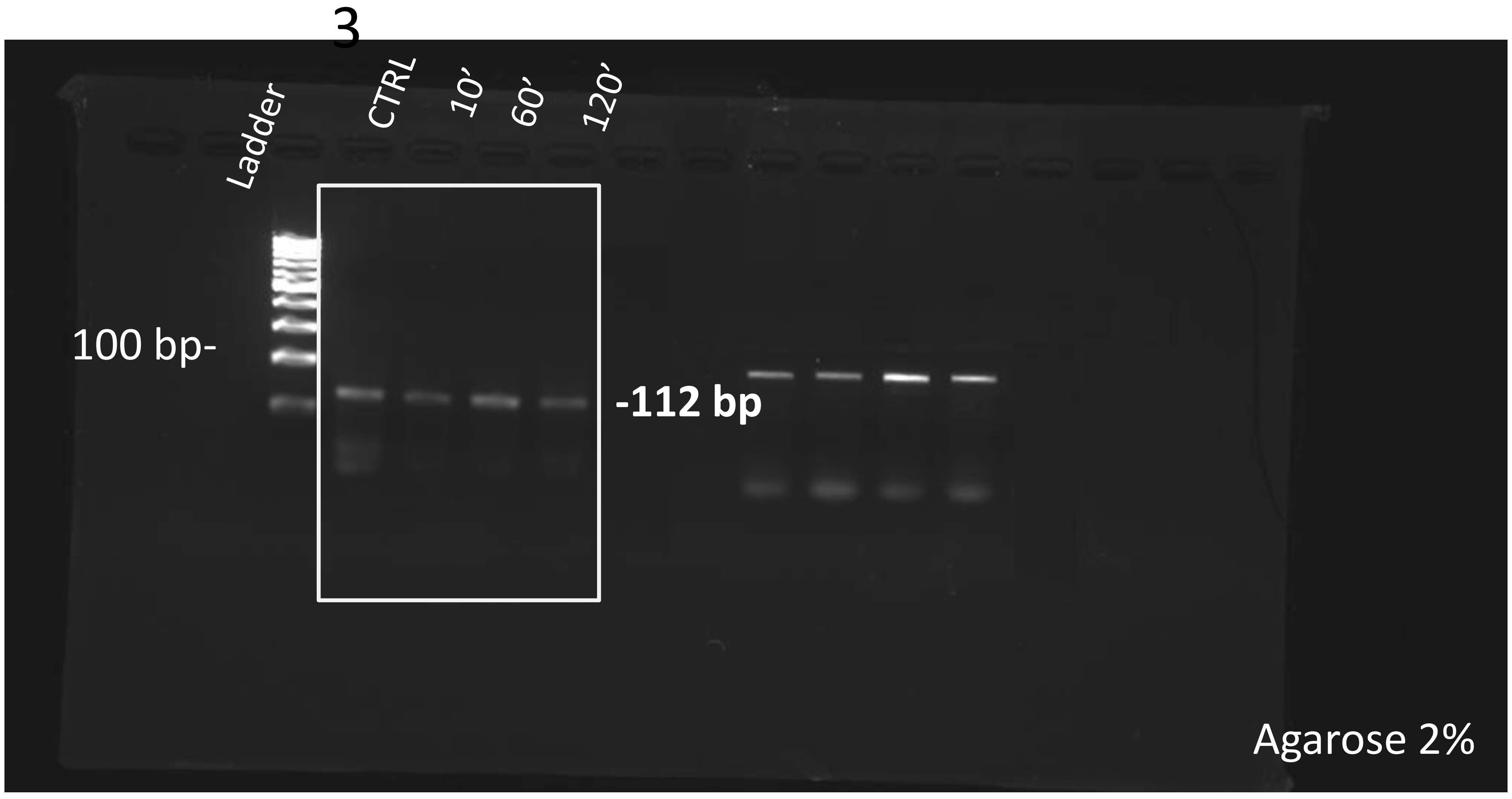

RT-PCR Tv+UV *tvrad51 assay* 1

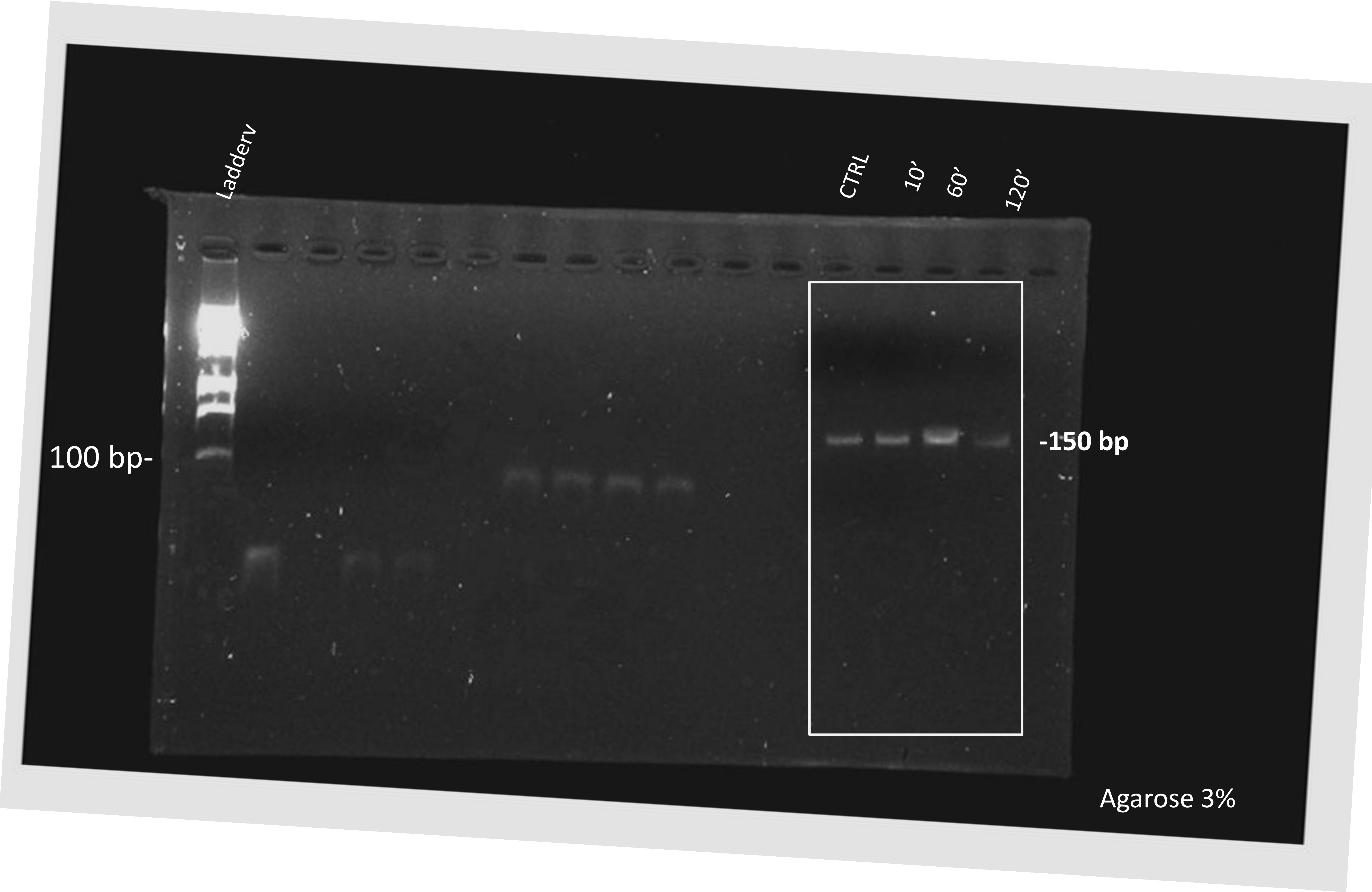


RT-PCR Tv+UV *tvrad51* assay *2*

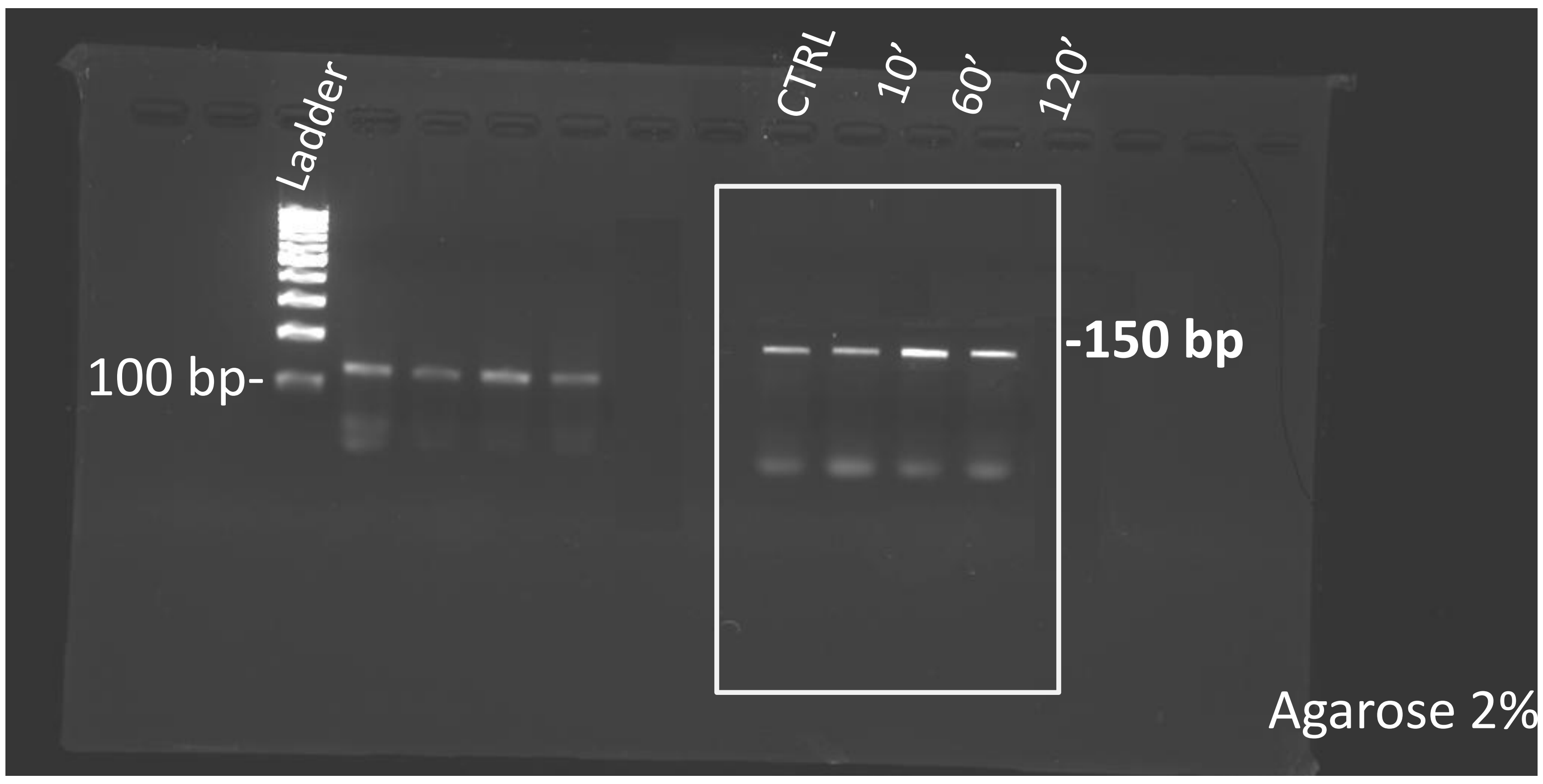


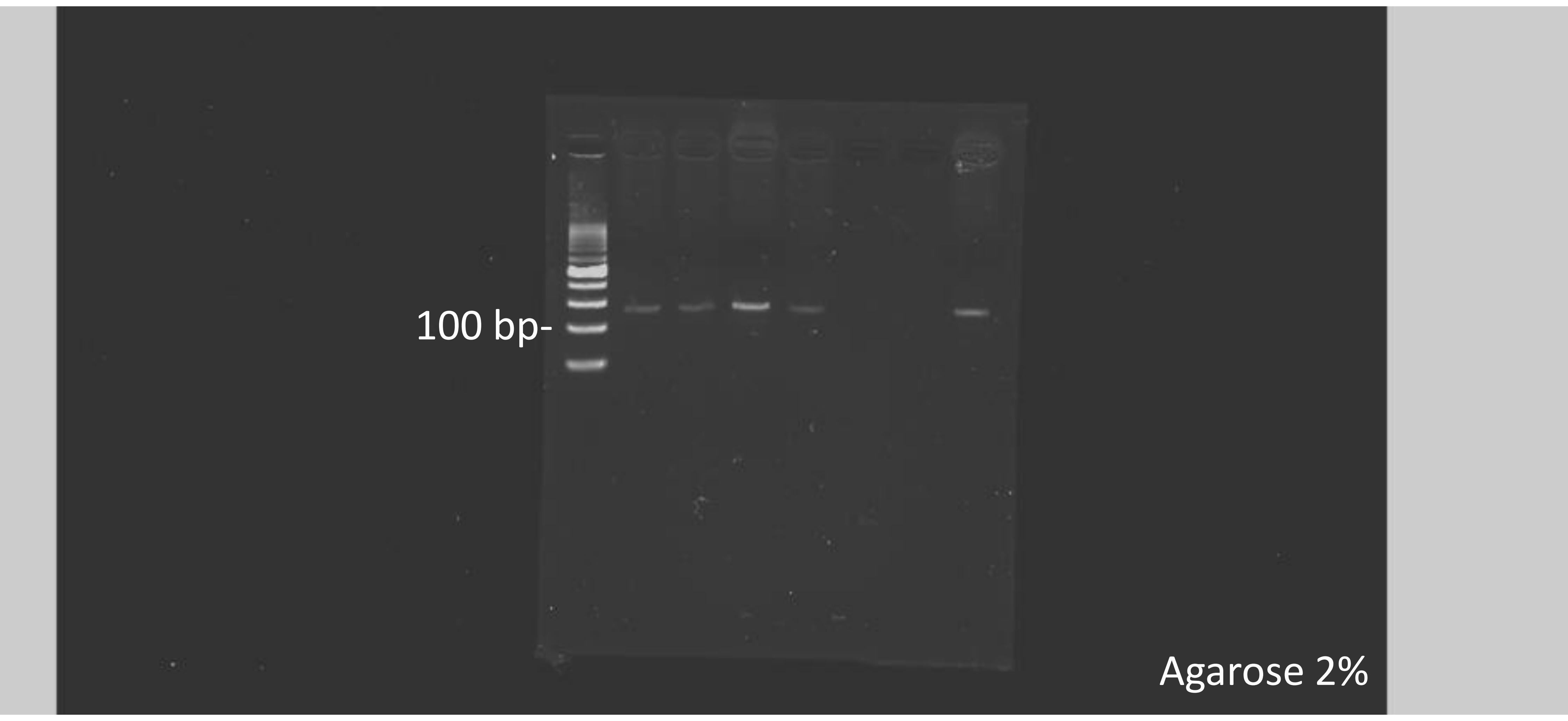


RT-PCR Tv+UV *tvrad51* assay 2

# RT-PCR Tv+$Zn^{2+}$ *tubulin 1*

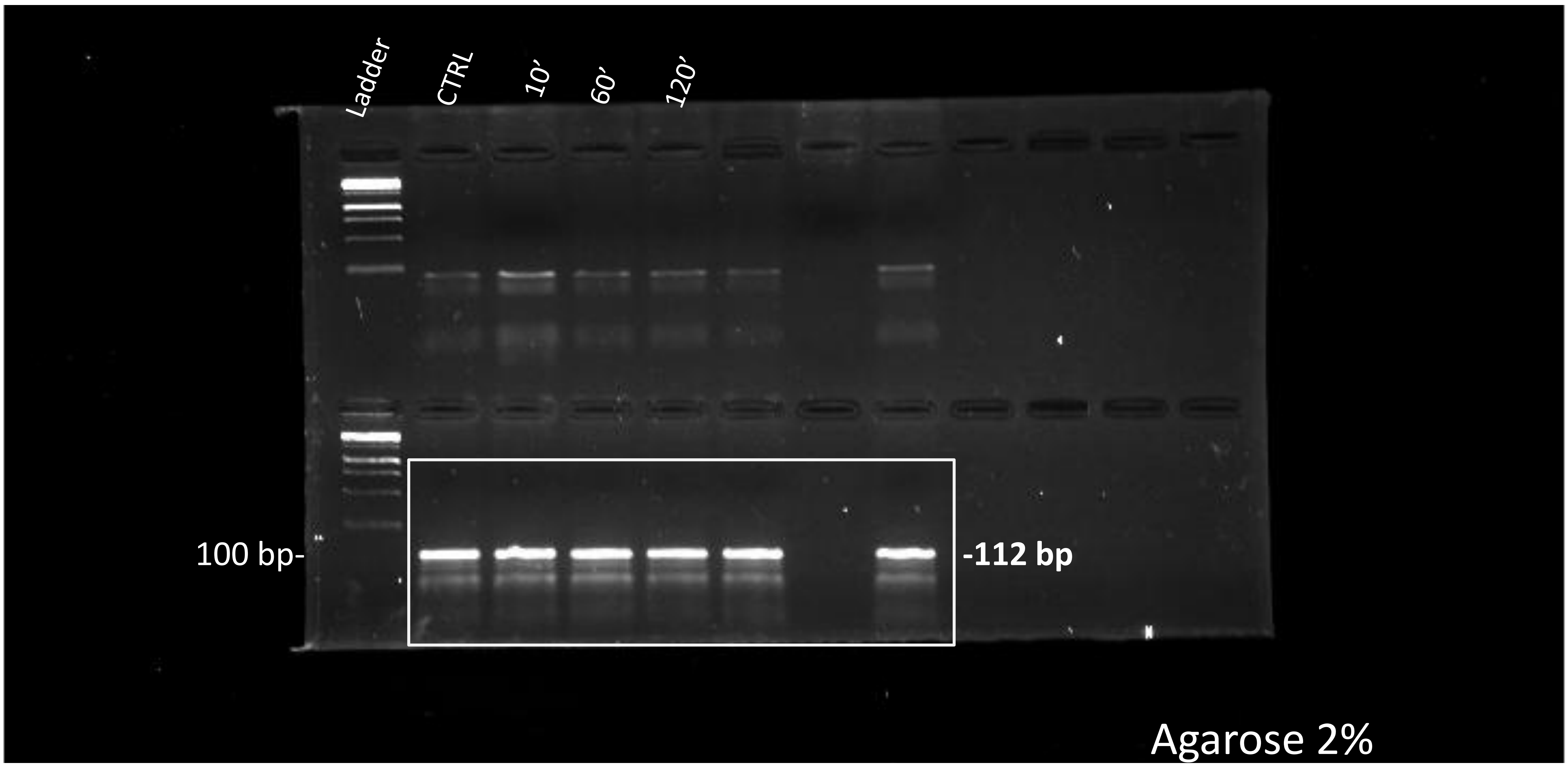


## RT-PCR Tv+$Zn^{2+}$ *tubulin 2*

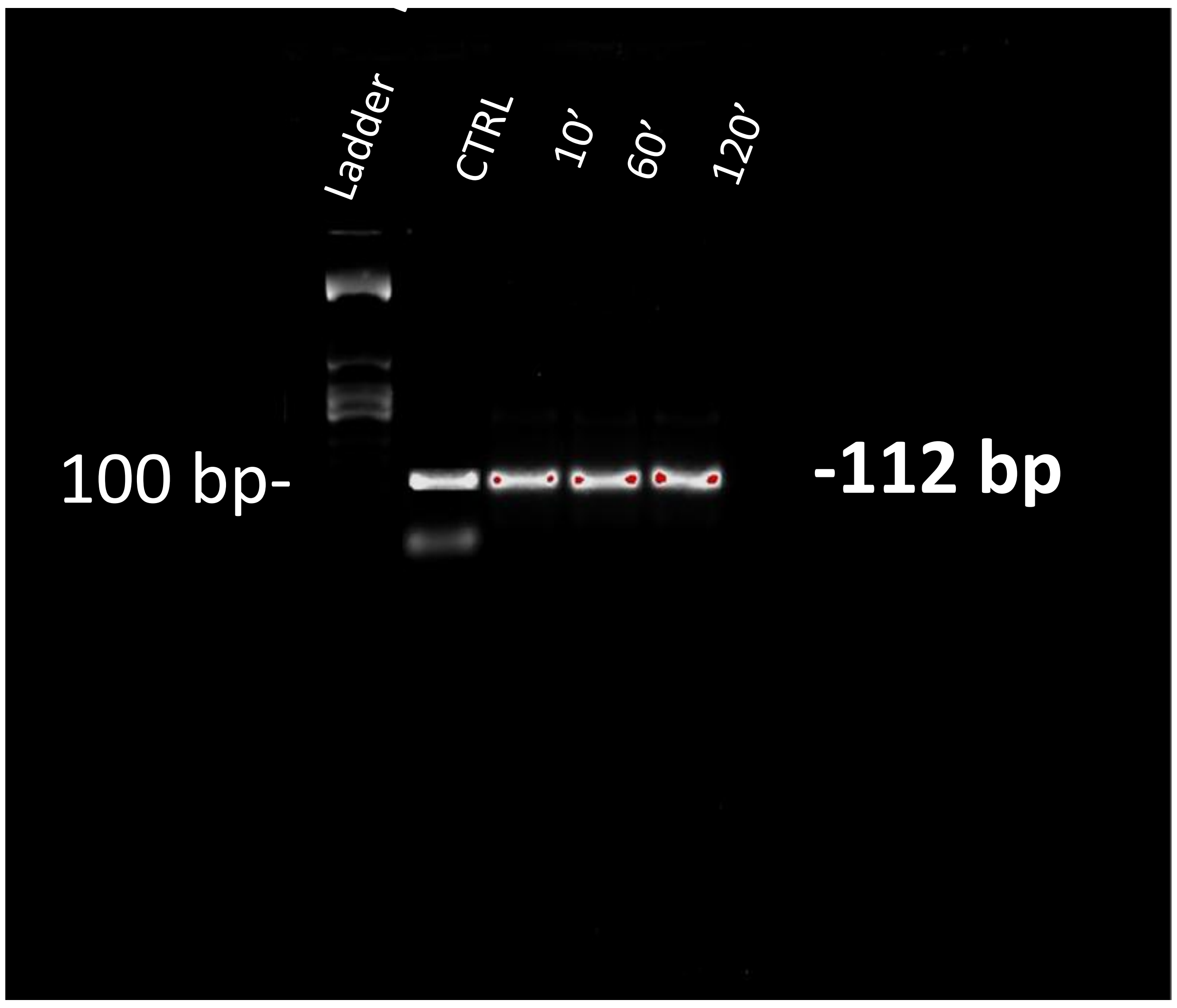


Agarose 2%

## RT-PCR Tv+$Zn^{2+}$ *tubulin 3*

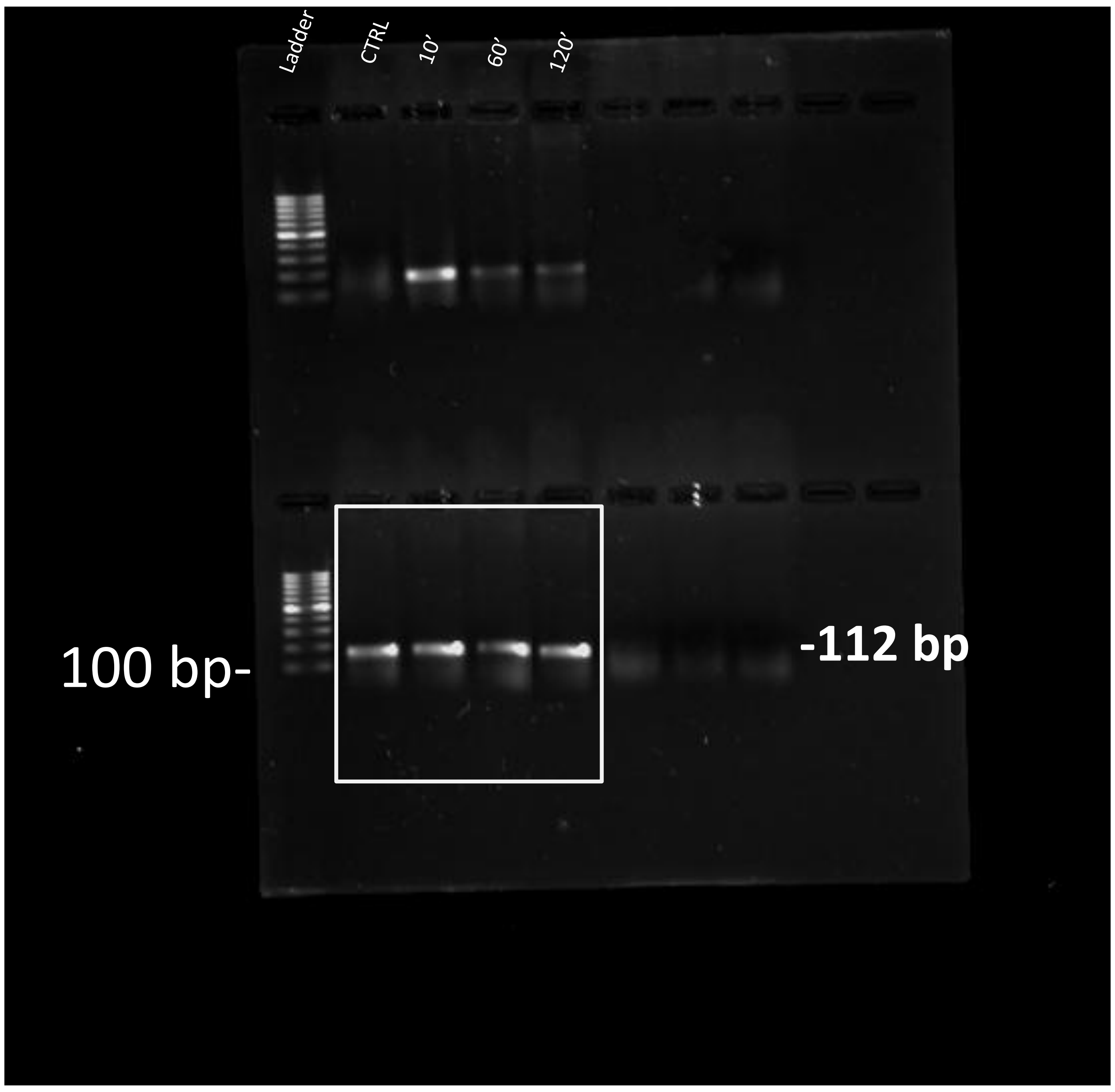


Agarose 2%

RT-PCR Tv+$Zn^{2+}$ *tvrad51* assay 1

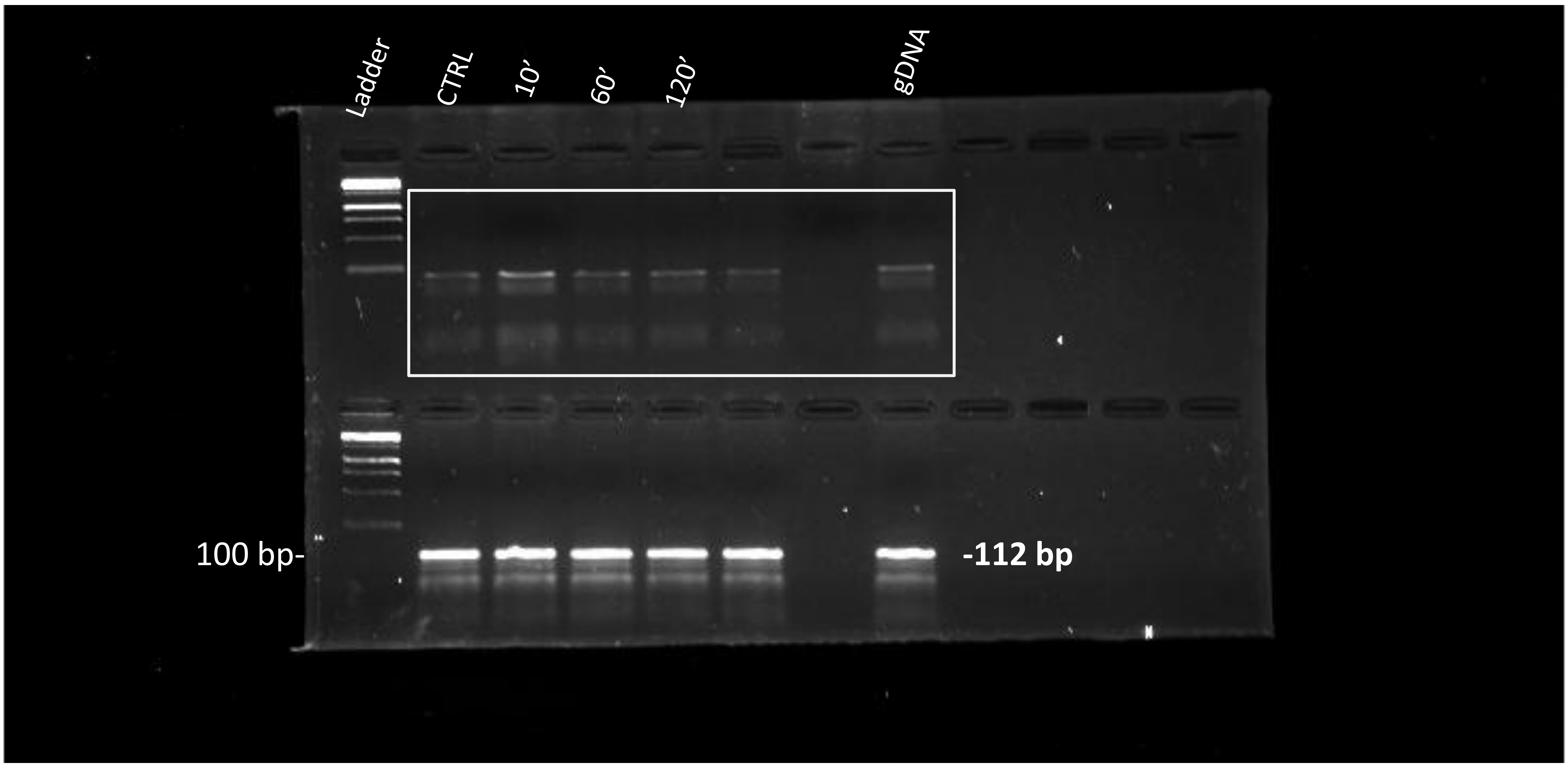


RT-PCR Tv+$Zn^{2+}$ *tvrad51* assay 2

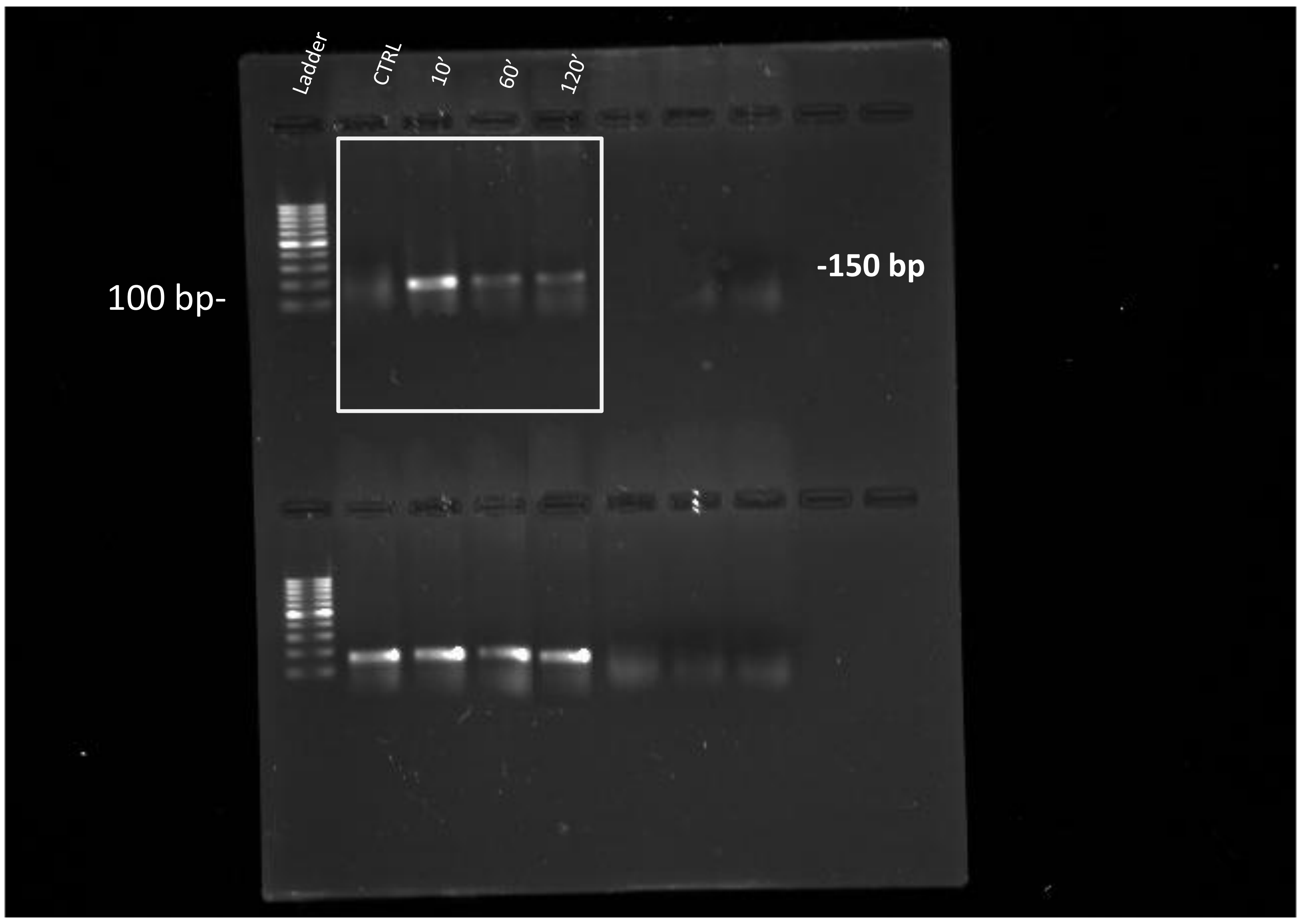


RT-PCR Tv+$Zn^{2+}$ *tvrad51* assay 3

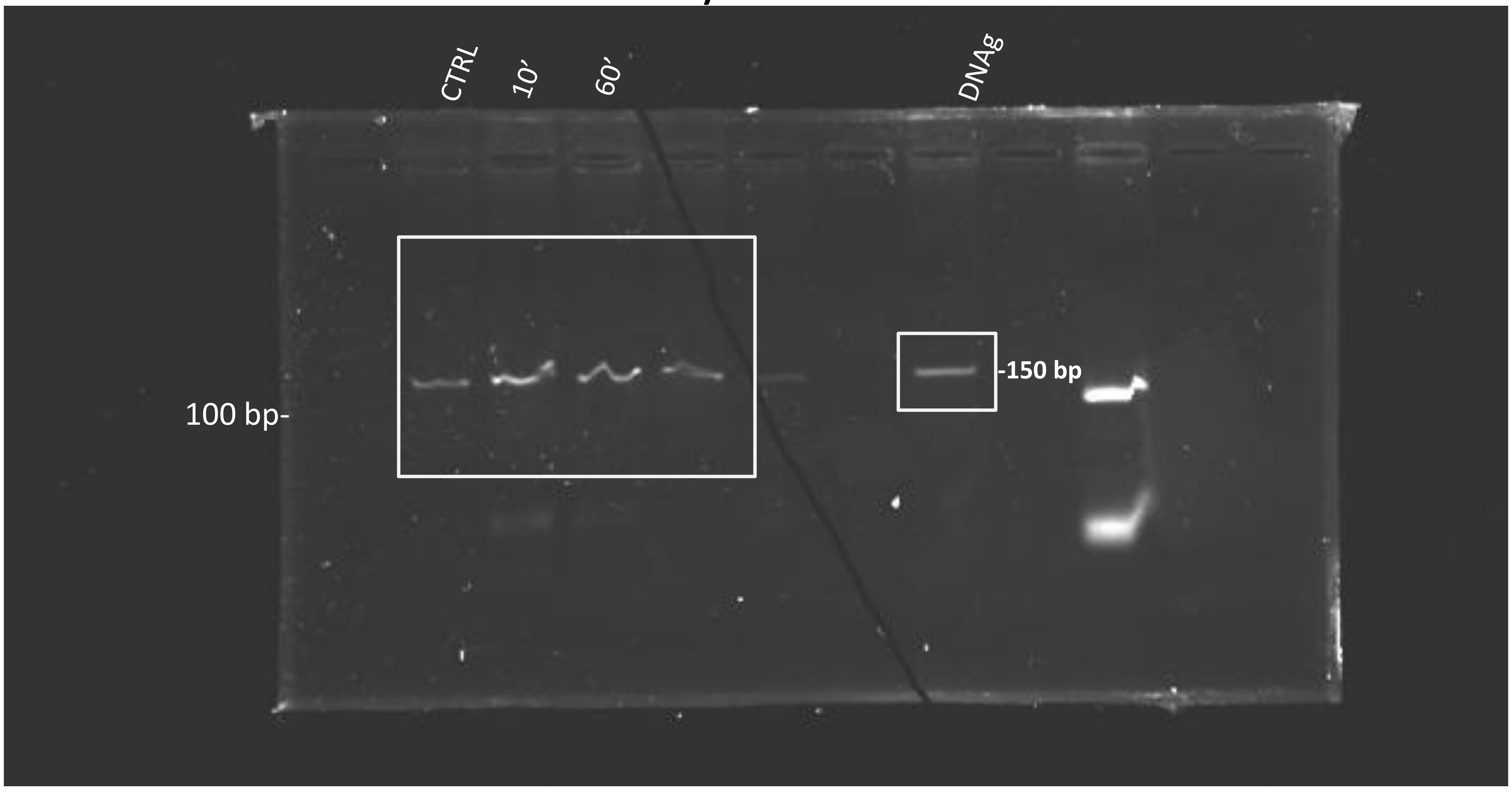

RT-PCR Tv+$Cd^{2+}$ *tubulin* assay *1*

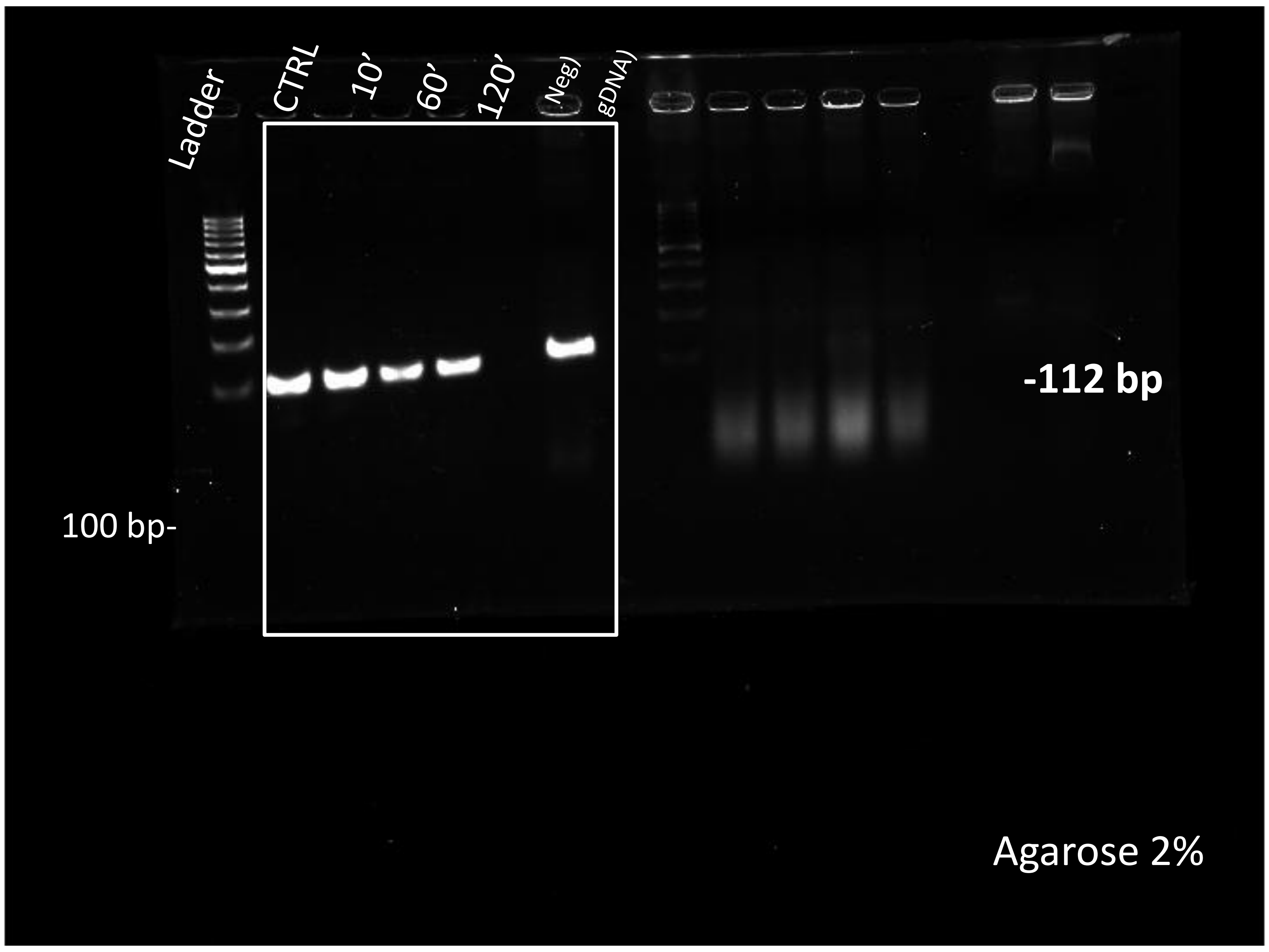


RT-PCR Tv+$Cd^{2+}$ *tubulin* assay 2

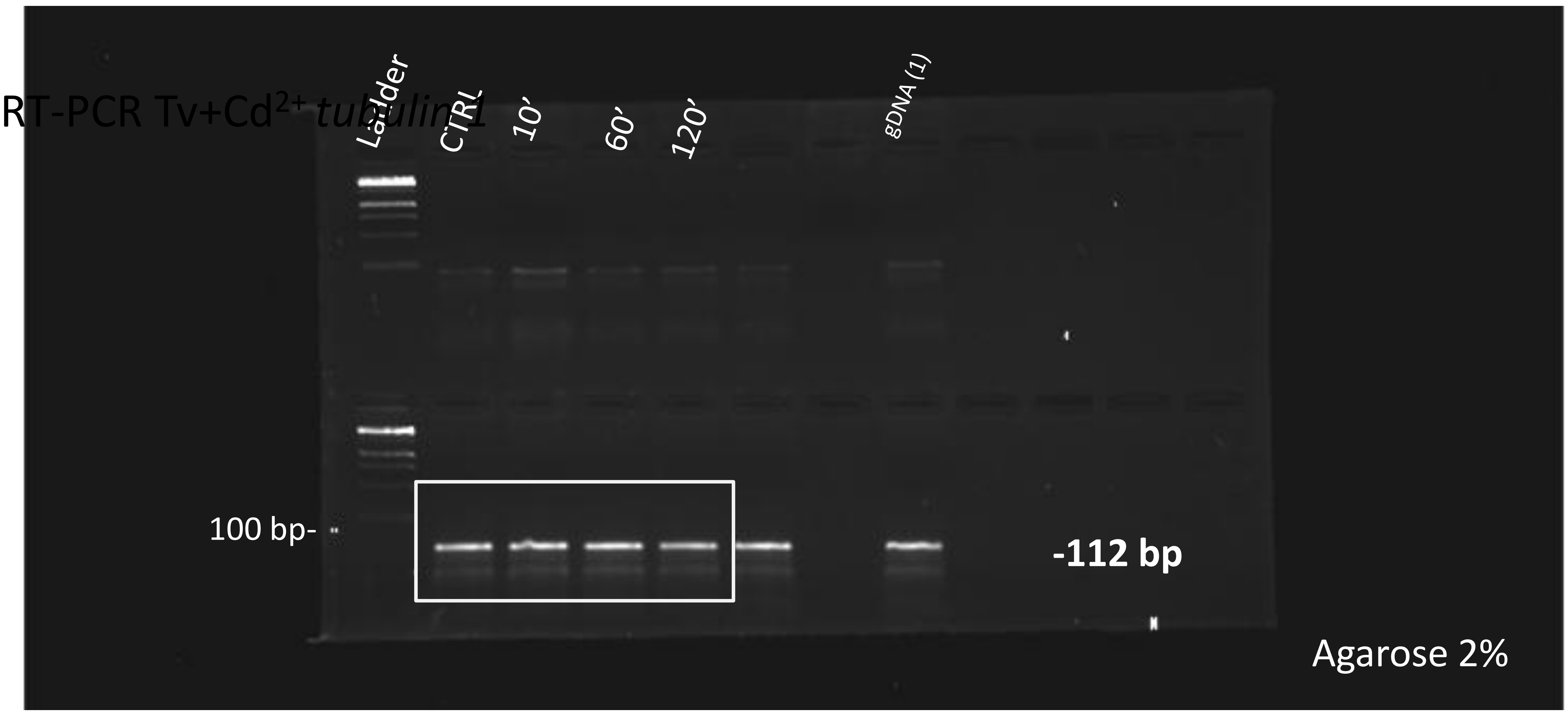


RT-PCR Tv+$Cd^{2+}$ *tubulin* assay 3

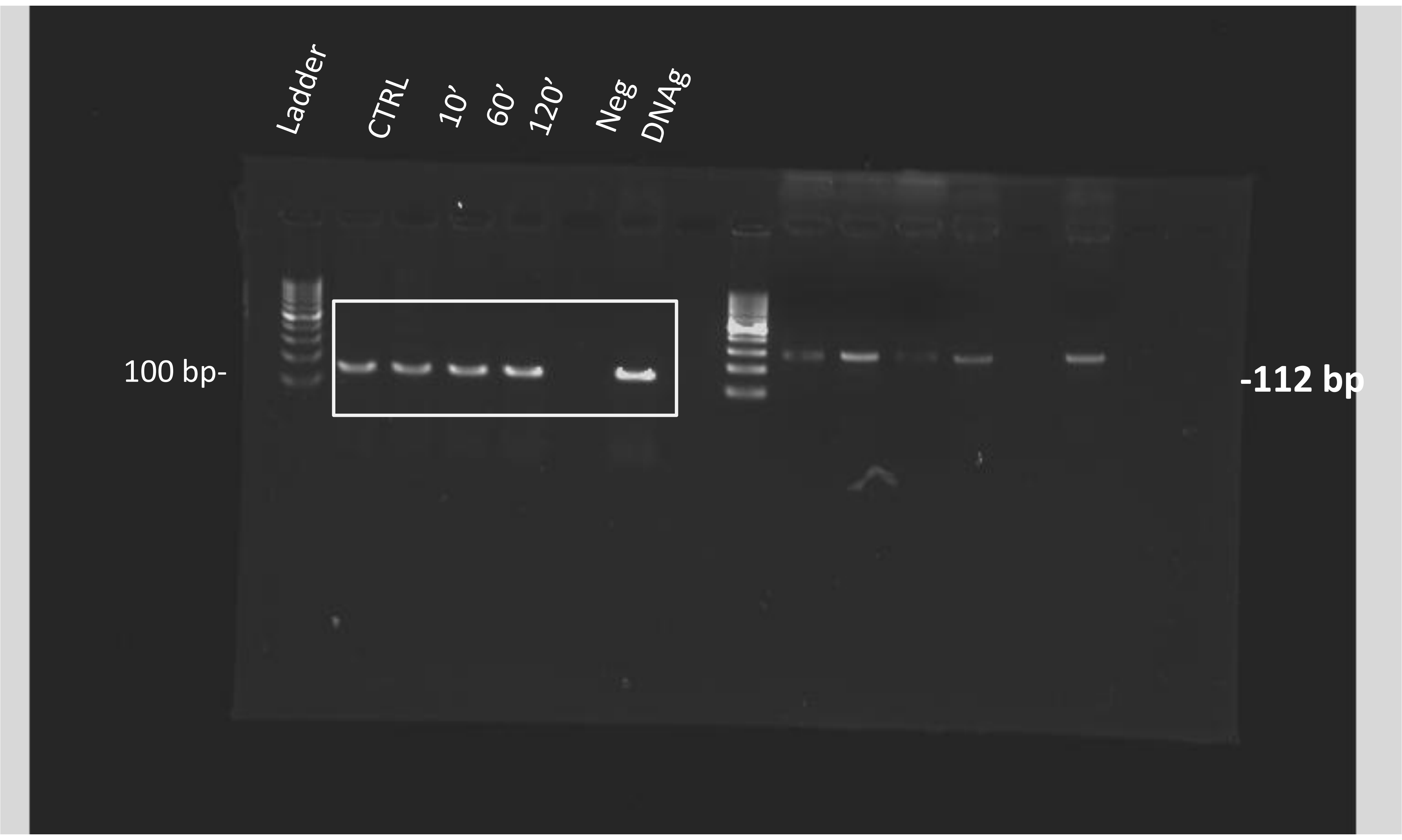

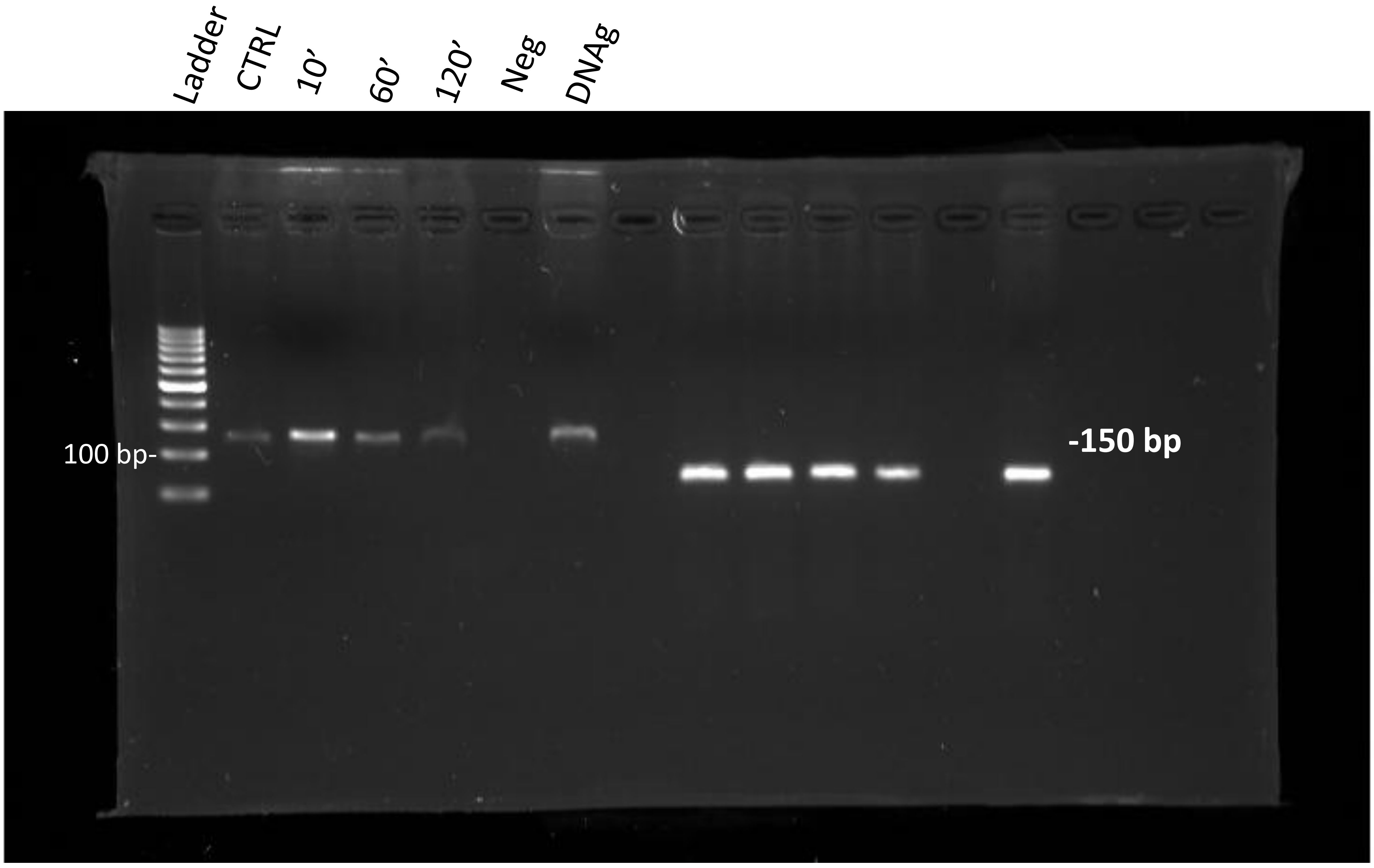


RT-PCR Tv+$Cd^{2+}$ *tvrad51* assay *1*

RT-PCR Tv+$Cd^{2+}$ *tvrad51* assay *2*

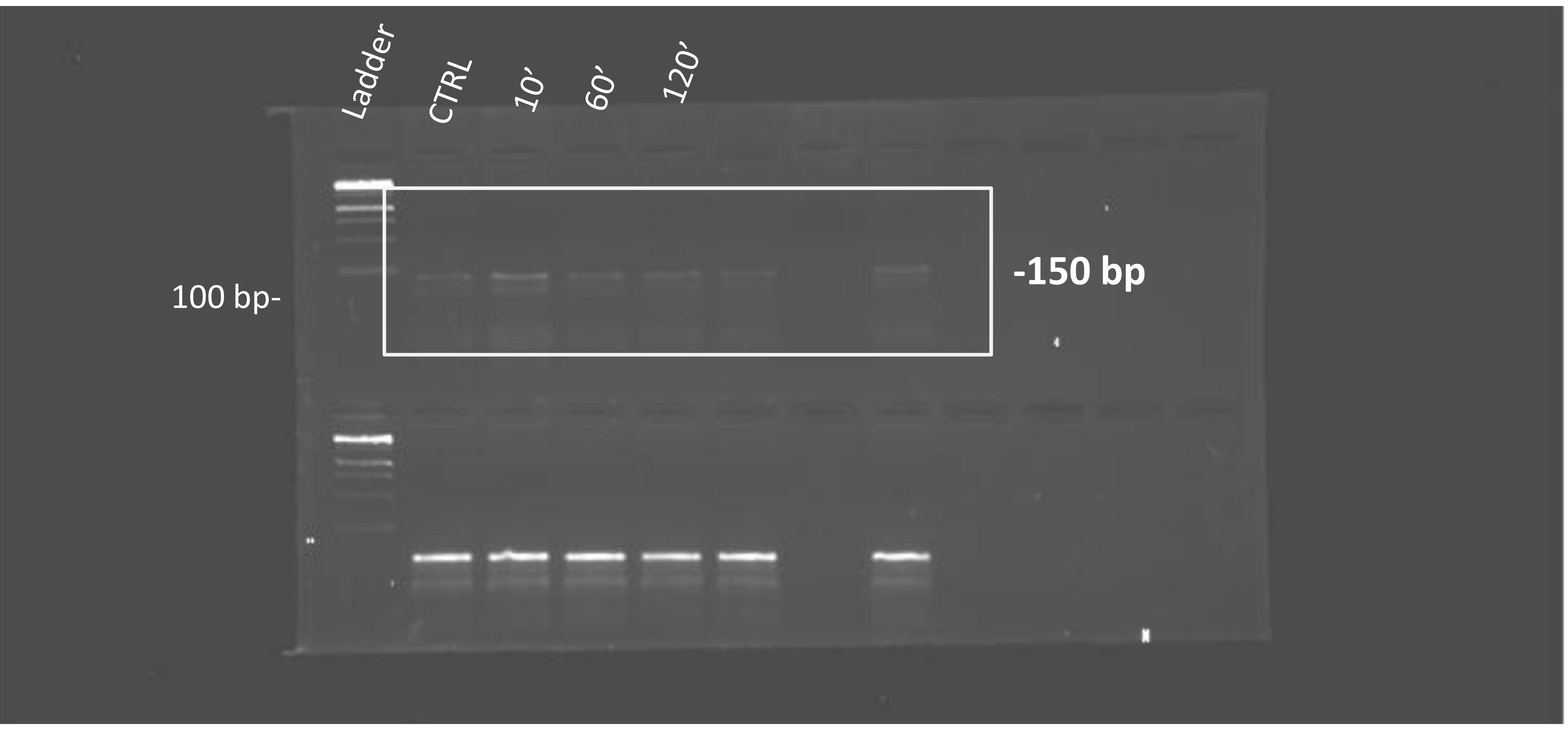


RT-PCR Tv+$Cd^{2+}$ *tvrad51* assay *2*

RT-PCR Tv+$Cd^{2+}$ *tvrad51* assay *3*

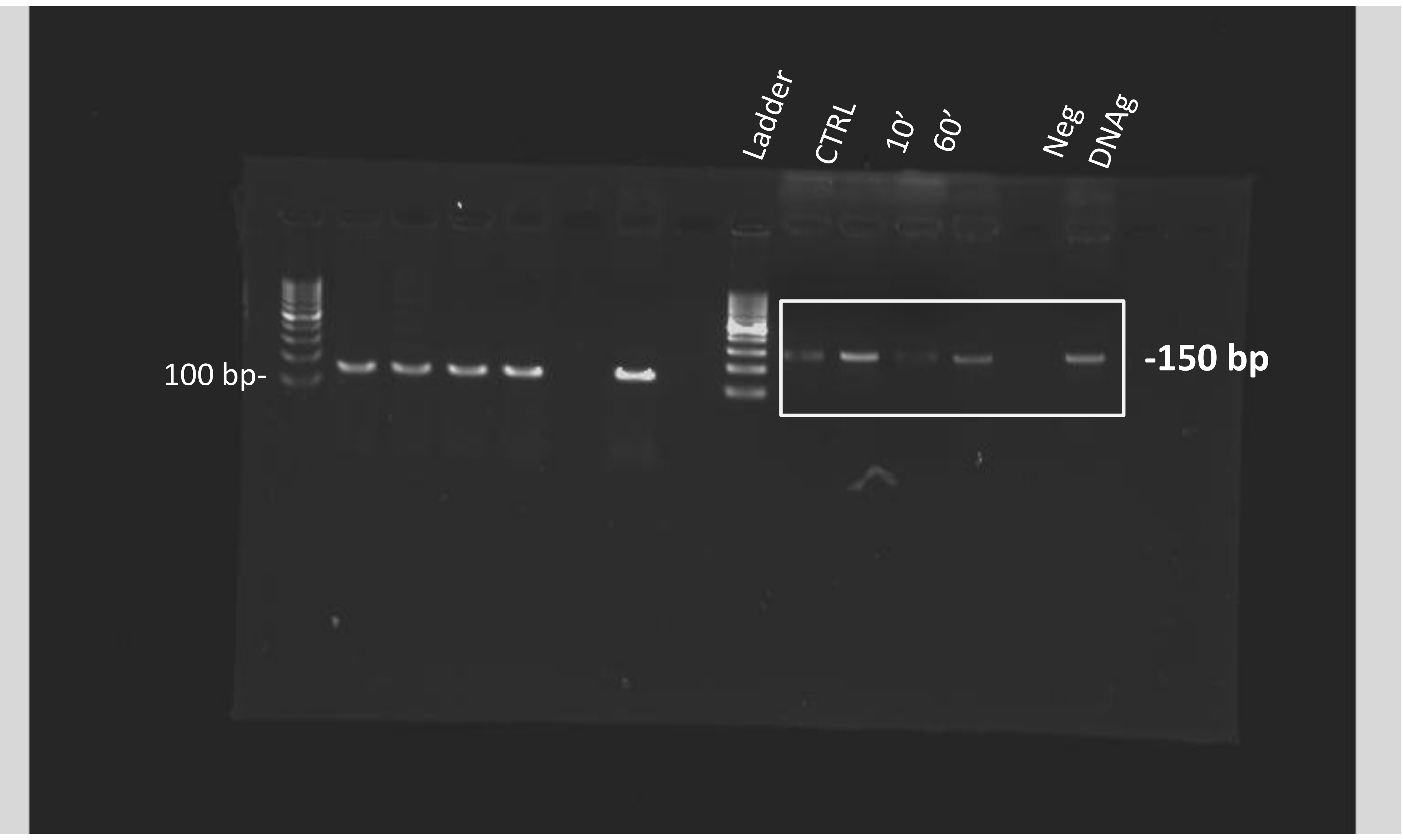

Supplement: Supplementary file 1 [file pathogens-14-00565-s001.zip › Figure S1..pdf]
