# Supplementary material for: Modulation of TvRAD51 Recombinase in Trichomonas vaginalis by Zinc and Cadmium as a Potential Mechanism for Genotoxic Stress Response
Source: Pathogens. 2025 Jun 5;14(6):565. doi: 10.3390/pathogens14060565 (PMC12195773; doi:10.3390/pathogens14060565)
Supplement: Supplementary file 1 [file pathogens-14-00565-s001.zip › Figure S2..pdf]

Figure S2. Images of IFA assays of *T. vaginalis* under different genotoxic conditions.

Trichomonas vaginalis **Zn^{2+} 10 min exposure**

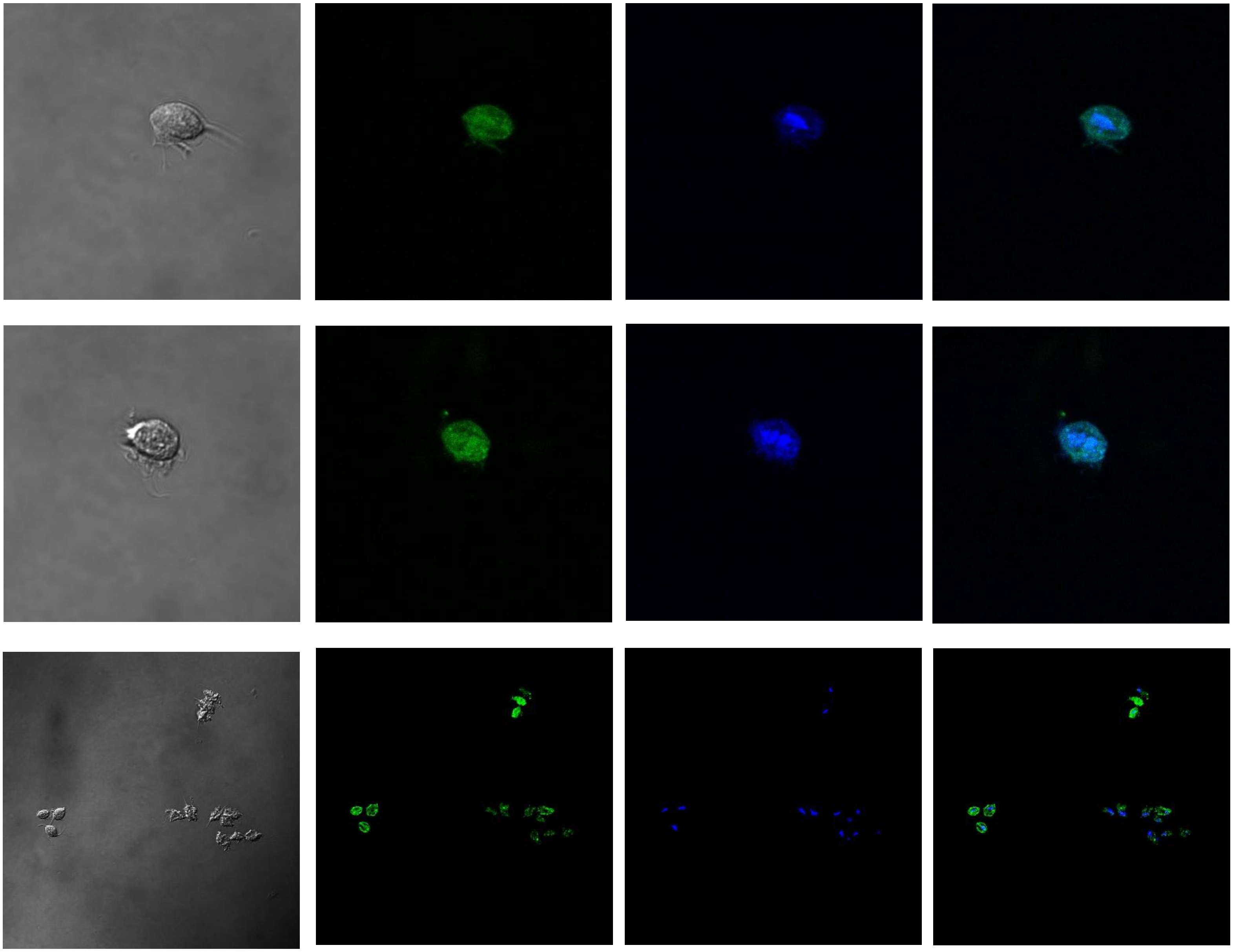

Trichomonas vaginalis Zn^{2+} 60 min exposure

Brightfield	DAPI	TvRAD51 (FITC)	Merged

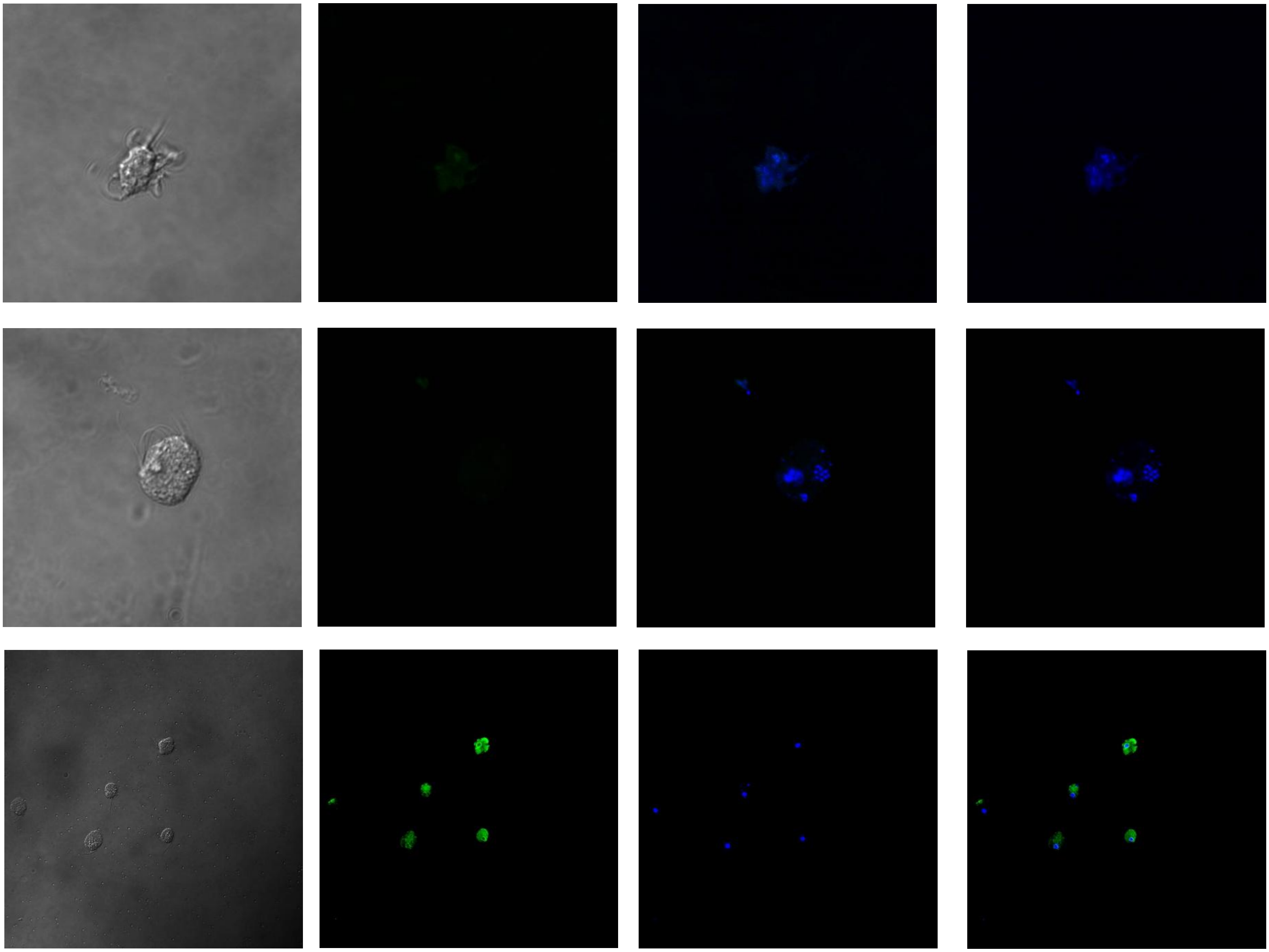

*Trichomonas vaginal*is Zn^{2+} 120 min exposure

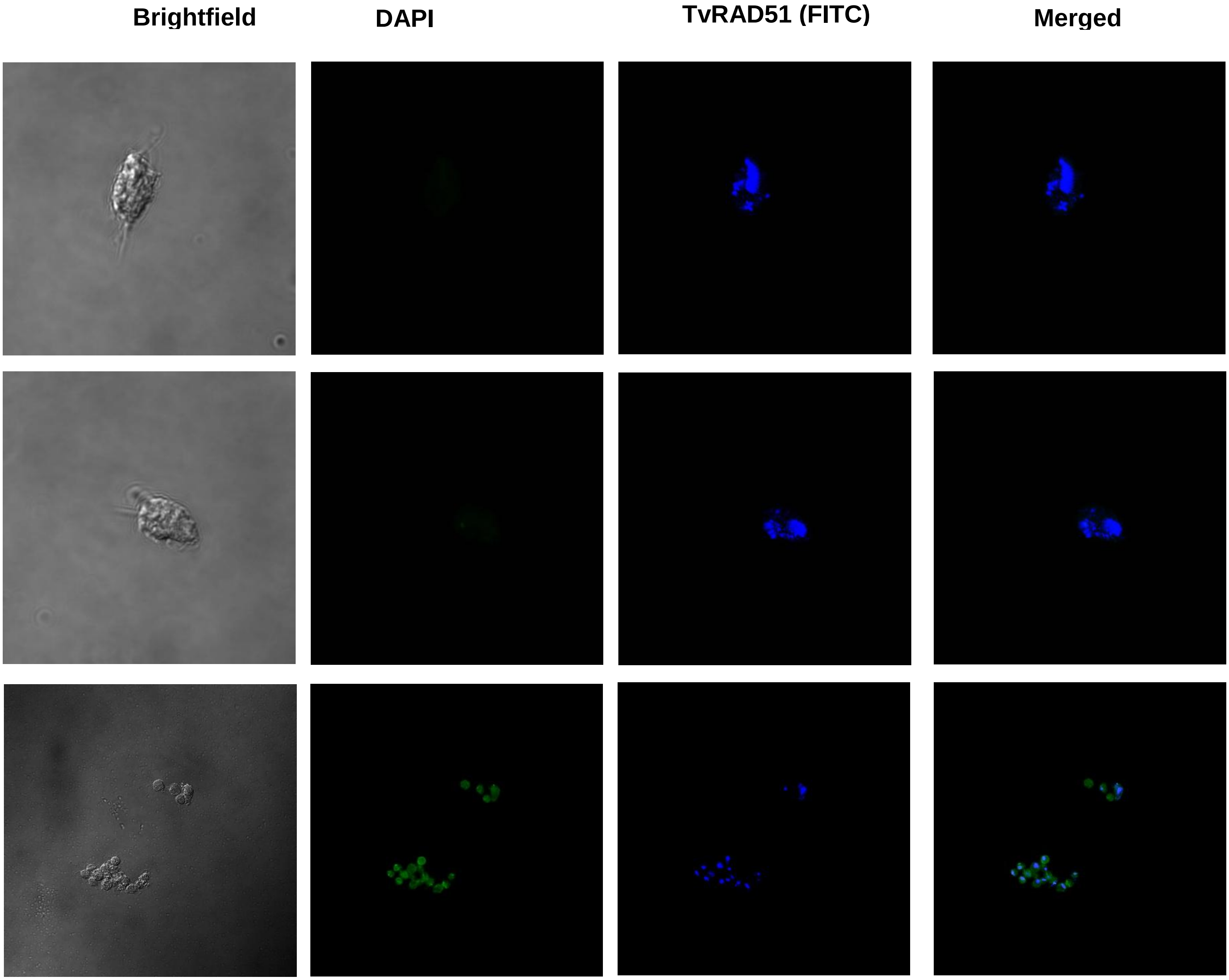

*Trichomonas vaginal*is Cd^{2+} 10 min exposure

Brightfield

TvRAD51 (FITC)

DAPI

Merged

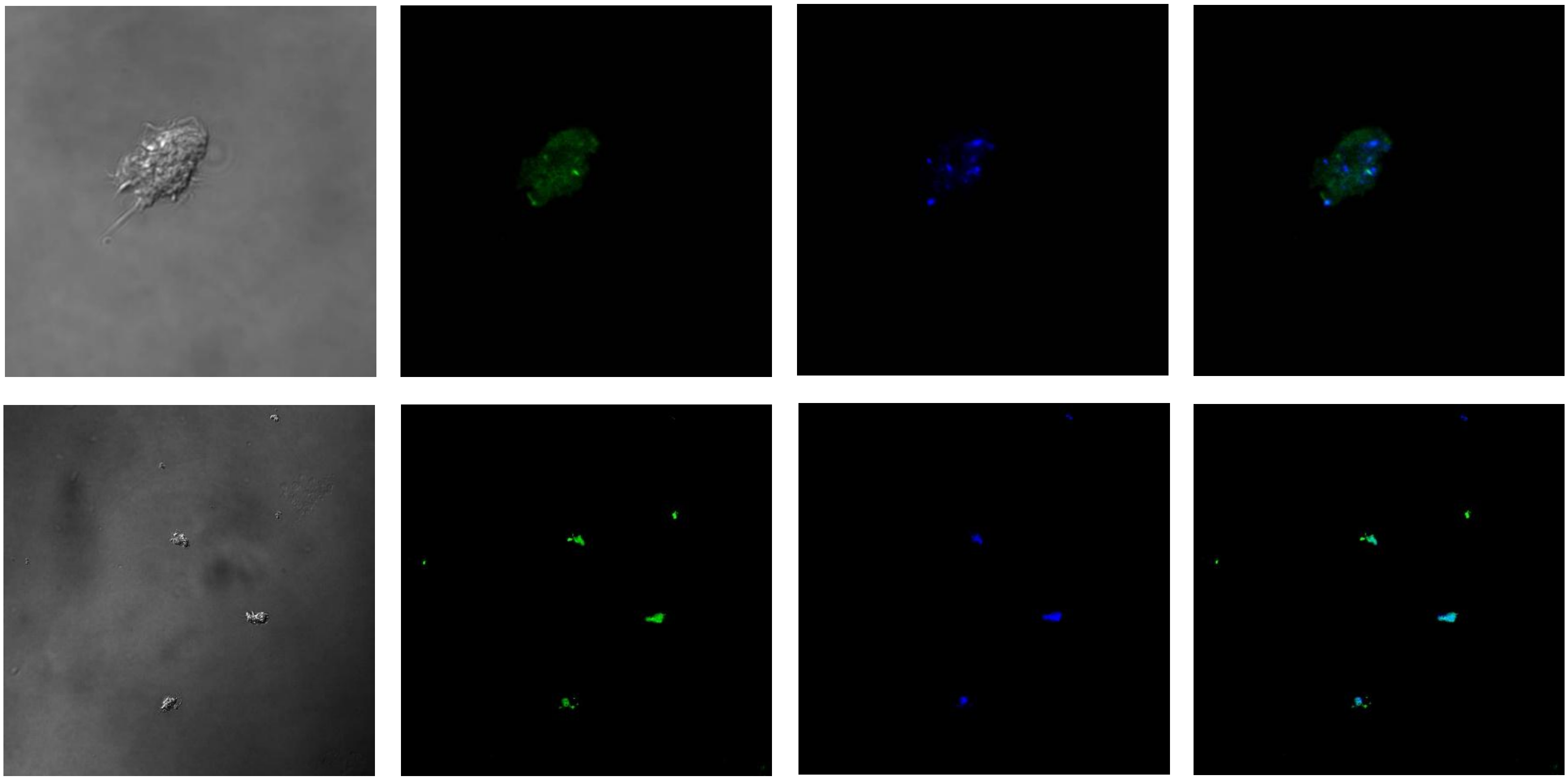

*Trichomonas vaginal*is Cd^{2+} 60 min exposure

TvRAD51FITC

DAPI

Merged

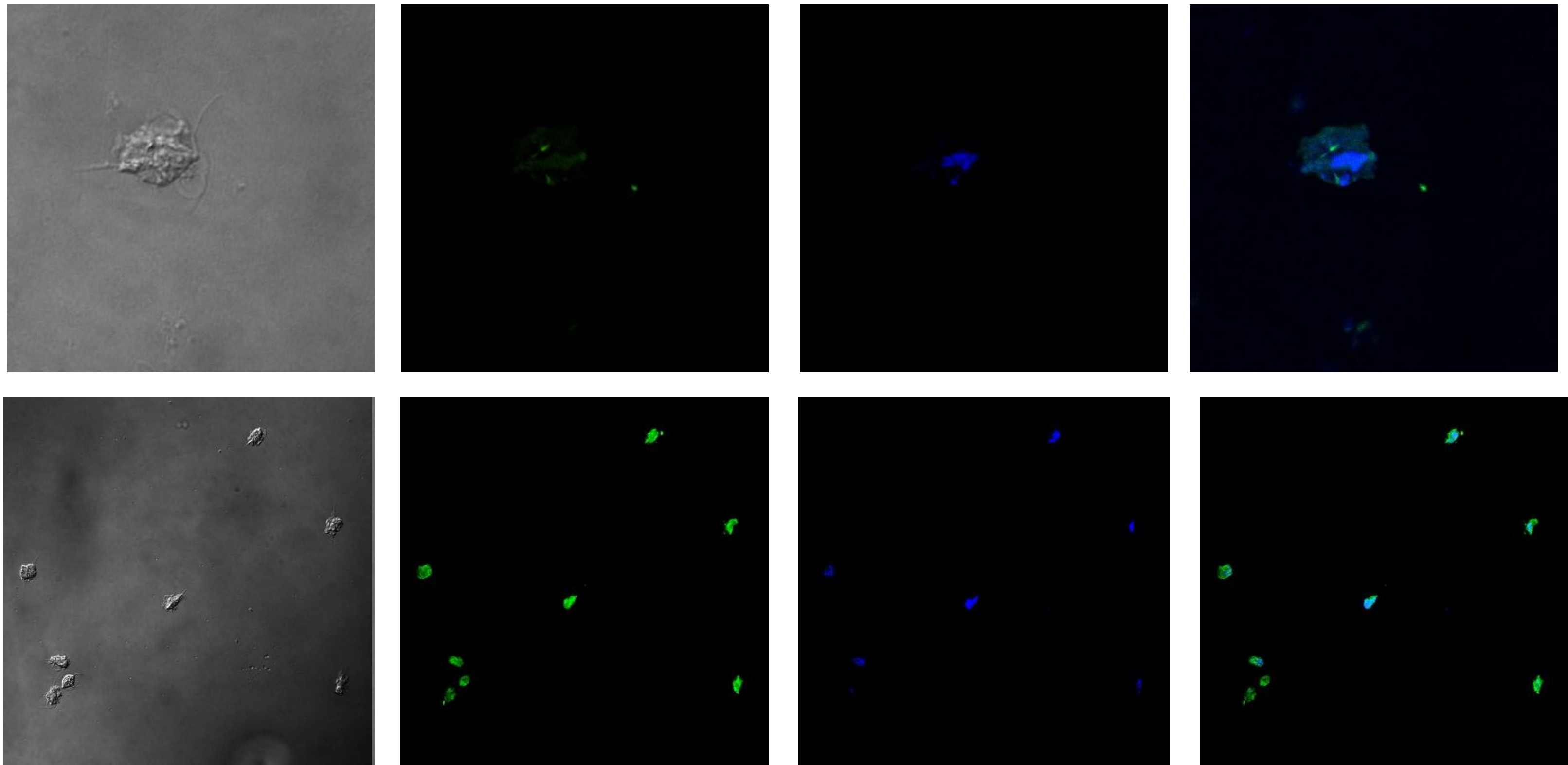

***Trichomonas vaginal*is Cd^{2+} 120 min exposure**

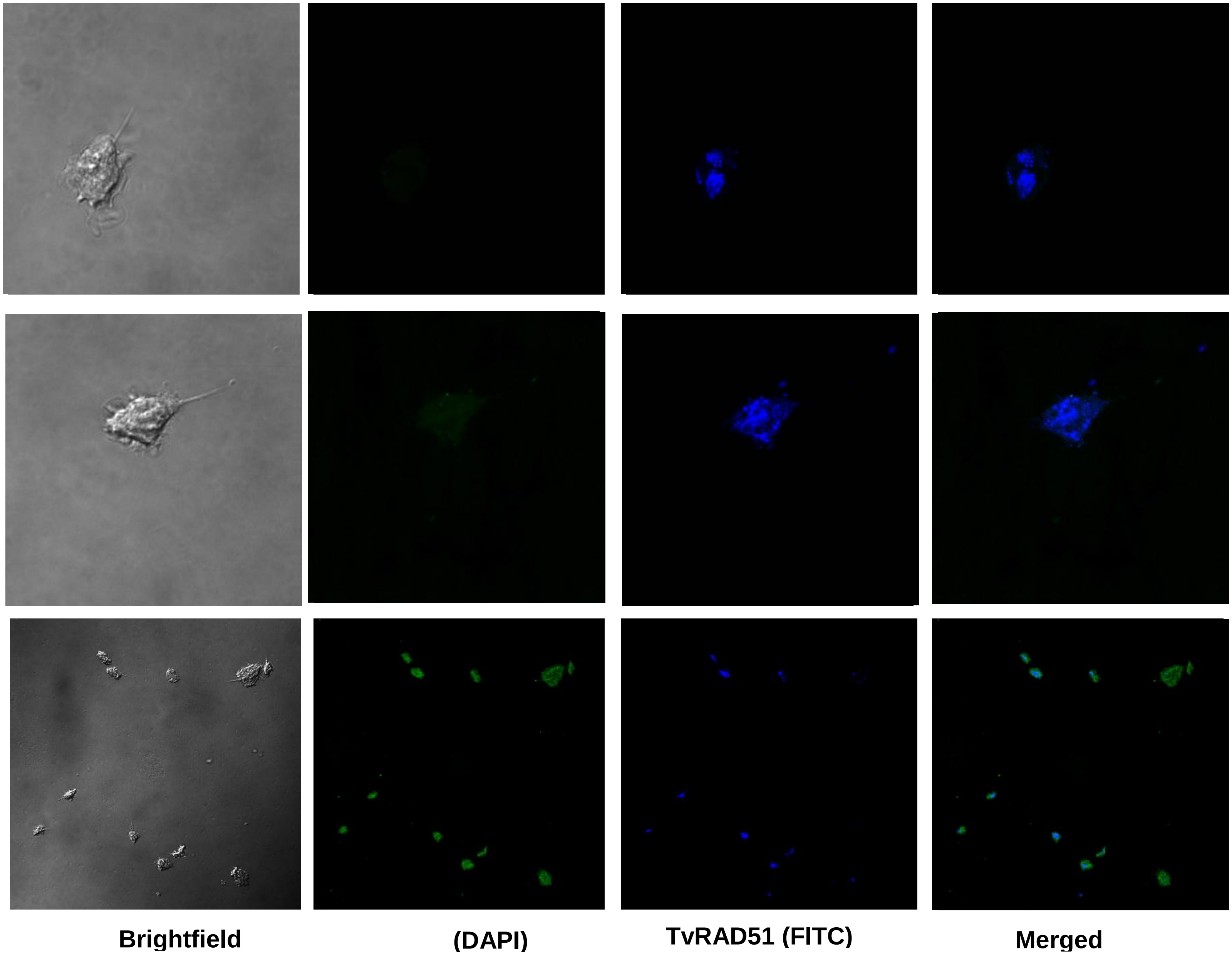
Brightfield
(DAPI)
TvRAD51 (FITC)
Merged

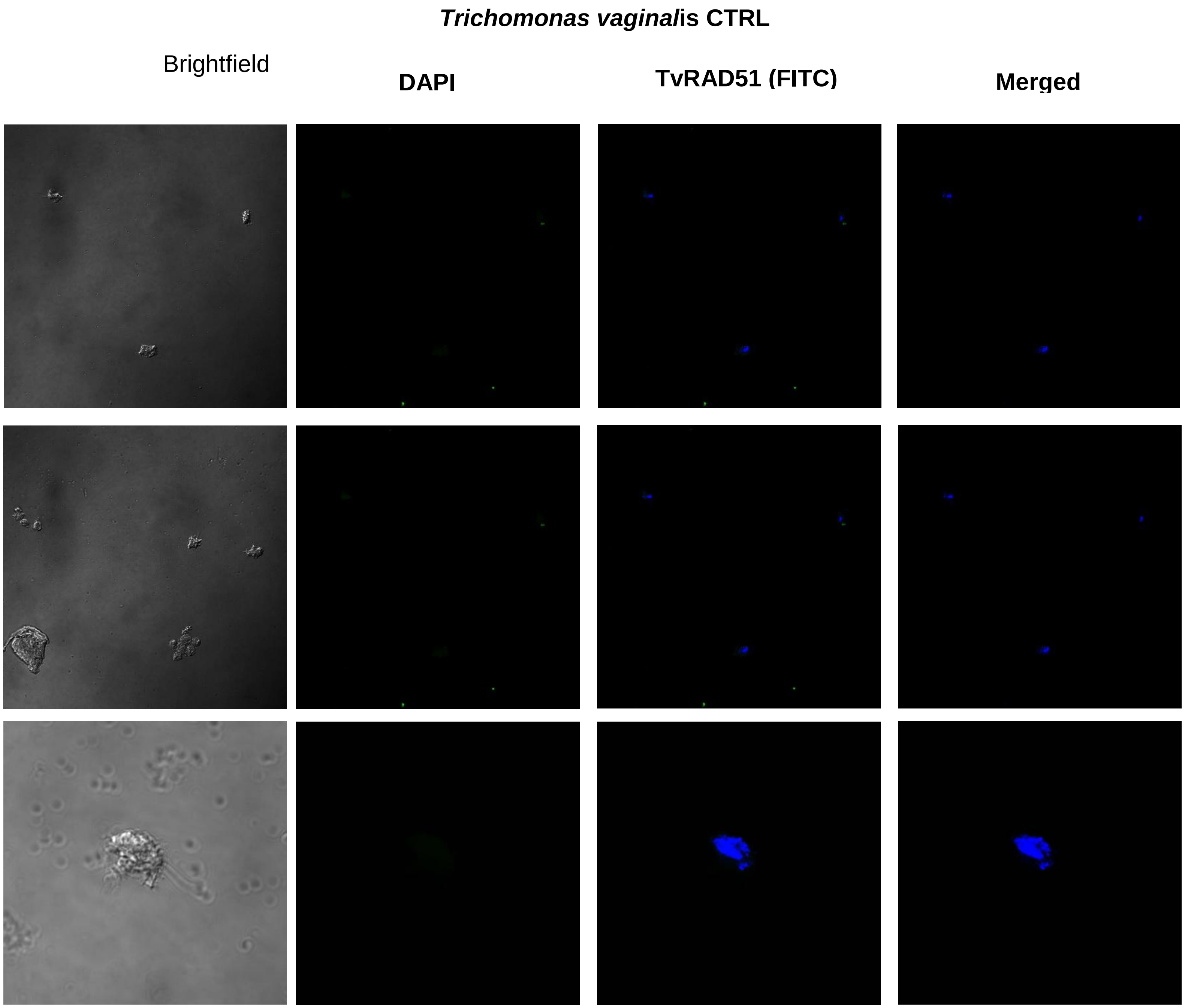

*Trichomonas vaginal*is CTRL
Brightfield
DAPI
TvRAD51 (FITC)
Merged
