# Supplementary material for: Modulation of TvRAD51 Recombinase in Trichomonas vaginalis by Zinc and Cadmium as a Potential Mechanism for Genotoxic Stress Response
Source: Pathogens. 2025 Jun 5;14(6):565. doi: 10.3390/pathogens14060565 (PMC12195773; doi:10.3390/pathogens14060565)
Supplement: Supplementary file 1 [file pathogens-14-00565-s001.zip › Figure S3.pdf]

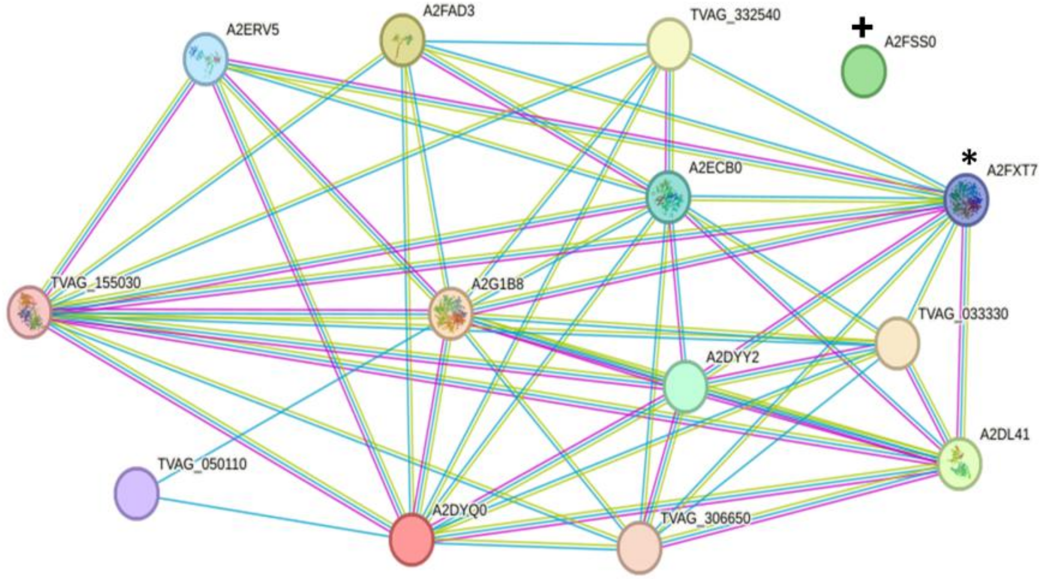

Figure S3. Protein–protein interaction cored network analysis with putative proteins of *T. vaginalis* HR machinery. (A) The interaction between the proteins evidence is shown by network nodes denoted by color: colored nodes: query proteins and first shell of interactors, white nodes: second shell of interactors. Node Content empty nodes: proteins of unknown 3D structure, filled nodes: a 3D structure is known or predicted. The edges indicate that the proteins are part of a physical complex are represent with color lines and indicates different parameters as known Interactions from curated databases (blue lines), experimentally determined (magenta lines) and predicted interactions as gene neighborhood (green lines) and other as textmining with the follow network stats: number of nodes: 14, number of edges: 53, average node degree, 7.57, average local clustering coefficient: 0.784, expected number of edges: 6, PPI enrichment p-value: $< 1.0 \times 10^{-16}$. The access number indicates the following *T. vaginalis* proteins: Recombinase TvRAD51 [A2FXT7, marked with an asterisk (*)] and TvRAD54B [A2FSS0 marked with a plus symbol (+)], TvBLM domain protein (A2DYY2), TvMRE11 protein domain (A2ECB0), TvRAD51D (A2DYQ0), TvRPA (A2DL41), TvRAD50 (A2FAD3), TvBRCA2 (A2ERV5), TvRAD54B (A2FSS0) and predicted physical partners TVAG_050110, TVAG_155030, TVAG_306650 and TVAG_033330.

Input

1) 'A2FXT7':

TVAG_204070 - DNA repair protein RAD51 homolog; Binds to single and double-stranded DNA and exhibits DNA- dependent ATPase activity. Underwinds duplex DNA. Belongs to the RecA family. RAD51 subfamily. *[a.k.a. 4747955, XP_001303202, DNA repair protein RAD51, A2FXT7]*

2) 'A2FSS0':

TVAG_441050 - SNF2 family N-terminal domain containing protein. *[a.k.a. 3.6.4.-, SNF2 family N-terminal domain containing protein, 4749749, A2FSS0]*

3) 'A2DYY2':

TVAG_255850 - ATP-dependent DNA helicase, RecQ family protein. *[a.k.a. 4772381, XM_001326581.1, ATP-dependent DNA helicase, RecQ family protein, A2DYY2]*

4) 'A2ECB0':

TVAG_098295 - Ser/Thr protein phosphatase, putative. *[a.k.a. XP_001321917, A2ECB0, Double-strand break repair protein MRE11]*

5) 'A2DYQ0':

TVAG_426330 - RECA_2 domain-containing protein. *[a.k.a. A2DYQ0, A2DYQ0_TRIVA, tva:TVAG_426330]*

6) 'A2DL41':

TVAG_294830 - Uncharacterized protein. *[a.k.a. 5464349, A2DL41, XP_001579818.1]*

7) 'A2FAD3':

TVAG_332600 - Zinc-hook domain-containing protein. *[a.k.a. 4755943, XP_001311080.1, A2FAD3]*

8) 'A2ERV5':

TVAG_473090 - BRCA2 repeat family protein. *[a.k.a. BRCA2 repeat family protein, tva:TVAG_473090, Breast cancer 2 susceptibility protein, A2ERV5]*

9) 'A2FSS0':

TVAG_441050 - SNF2 family N-terminal domain containing protein. *[a.k.a. 3.6.4.-, SNF2 family N-terminal domain containing protein, 4749749, A2FSS0]*

10) 'TVAG_155030':

TVAG_155030 - Meiotic recombination protein DMC1/LIM15 homolog, putative; Belongs to the RecA family.

11) 'TVAG_306650':

TVAG_306650 - DNA topoisomerase; Introduces a single-strand break via transesterification at a target site in duplex DNA. Releases the supercoiling and torsional tension of DNA introduced during the DNA replication and transcription by transiently cleaving and rejoining one strand of the DNA duplex. The scissile phosphodiester is attacked by the catalytic tyrosine of the enzyme, resulting in the formation of a DNA-(5'-phosphotyrosyl)-enzyme intermediate and the expulsion of a 3'-OH DNA strand. Belongs to the type IA topoisomerase family.

12) 'TVAG_033330':

☑ TVAG_033330 - DNA topoisomerase; Introduces a single-strand break via transesterification at a target site in duplex DNA. Releases the supercoiling and torsional tension of DNA introduced during the DNA replication and transcription by transiently cleaving and rejoining one strand of the DNA duplex. The scissile phosphodiester is attacked by the catalytic tyrosine of the enzyme, resulting in the formation of a DNA-(5'-phosphotyrosyl)-enzyme intermediate and the expulsion of a 3'-OH DNA strand. Belongs to the type IA topoisomerase family.
